# Urine lipoarabinomannan glycan in HIV-negative patients with pulmonary tuberculosis correlates with disease severity

**DOI:** 10.1126/scitranslmed.aal2807

**Published:** 2017-12-13

**Authors:** Luisa Paris, Ruben Magni, Fatima Zaidi, Robyn Araujo, Neal Saini, Michael Harpole, Jorge Coronel, Daniela E. Kirwan, Hannah Steinberg, Robert H. Gilman, Emanuel F. Petricoin, Roberto Nisini, Alessandra Luchini, Lance Liotta

**Affiliations:** 1 George Mason University, Manassas, VA 20110, USA; 2 Queensland University of Technology, Brisbane, Queensland 4000, Australia; 3 Universidad Peruana Cayetano Heredia, Lima 31, Peru; 4 St. George’s Hospital, London SW17 0QT, UK; 5 Johns Hopkins University, Baltimore,MD21205, USA; 6 6Istituto Superiore di Sanità, Rome 00161, Italy

## Abstract

An accurate urine test for pulmonary tuberculosis (TB), affecting 9.6 million patients worldwide, is critically needed for surveillance and treatment management. Past attempts failed to reliably detect the mycobacterial glycan antigen lipoarabinomannan (LAM), a marker of active TB, in HIV-negative, pulmonary TB–infected patients’ urine (85% of 9.6 million patients). We apply a copper complex dye within a hydrogel nanocage that captures LAM with very high affinity, displacing interfering urine proteins. The technology was applied to study pretreatment urine from 48 Peruvian patients, all negative for HIV, with microbiologically confirmed active pulmonary TB. LAM was quantitatively measured in the urine with a sensitivity of >95%and a specificity of >80% (*n* = 101) in a concentration range of 14 to 2000 picograms per milliliter, as compared to non-TB, healthy and diseased, age-matched controls (evaluated by receiver operating characteristic analysis; area under the curve, 0.95; 95% confidence interval, 0.9005 to 0.9957). Urinary LAM was elevated in patients with a higher mycobacterial burden (*n* = 42), a higher proportion of weight loss (*n* = 37), or cough (*n* = 50). The technology can be configured in a variety of formats to detect a panel of previously undetectable very-low-abundance TB urinary analytes. Eight of nine patients who were smear-negative and culture-positive for TB tested positive for urinary LAM. This technology has broad implications for pulmonary TB screening, transmission control, and treatment management for HIV-negative patients.

## INTRODUCTION

An accurate screening test for active pulmonary tuberculosis (TB) is urgently needed for patients who are not coinfected with HIV (*1**,* *2*). Worldwide, TB is one of the most prevalent bacterial infections (9.6millioncasesand1.5milliondeaths in2014),withthehighestmortality in developing countries (*1*). Ideally, such a test would use a noninvasive body fluid such as urine to facilitate utilization in a low-resource setting (*1*, *2*). This objective, at first, appears straightforward because the outer surface glycan lipoarabinomannan (LAM), a TB antigen shed into the urine during active TB, has been identified and well characterized (3, 4). Although enzyme-linked immunosorbent assay and lateral flow tests have been developed to measure LAM, their sensitivity is limited (*4*). These tests can detect urinary LAM in patients with pulmonary TB who are coinfected with HIV (*5*, *6*) but not in those who are HIV-negative (*5*, *6*). Quantitative gas chromatography–mass spectrometry has been used to identify D-arabinose as a proxy for LAM in TB patients irrespective of HIV status, but the sensitivity is limited to 10 to 40 ng/ml (*7*). Unfortunately, the failure to detectLAMin the urine ofHIV-negative patients limits the applicability of these assays, because most of the TB patients areHIV-negative (85% of the 9.6million patients worldwide) (*1*).

The poor sensitivity of existing LAM assays in HIV-negative/ TB-positive patients has been explained by three main hypotheses.The first hypothesis is that LAM is shed into the urine of active pulmonary TB patients only in the context of glomerular dysfunction caused by HIV infection including HIV-related nephropathy (HIVAN) (*8*, *9*). However, in HIVAN, urinary LAMis not associated with heavy proteinuria, suggesting that this is not an importantmechanism (*9*). The second hypothesis is that LAM is shed into the urine of patients with active TB only when there is extrapulmonary renal tract involvement, such that the antigen can enter the urine directly from infected tissue (*10*). The third hypothesis is that the concentration of LAM in patients with active pulmonary TB is below the concentration detection limits of existing assays and may be masked by formation of immune complexes, excess non-LAM proteins, or other inhibitors present in the urine (*7*). Here, we apply a new class of analytical nanocage technology to definitively address these hypotheses and solve this dilemma.

The ideal urine test would measure a variety of pathogen and host analytes (*2*) to achieve the highest specificity and accuracy at all phases of TB. Therefore, we explored whether the nanocage technology could be extended to other characterized analytes associated with TB and the host response and to other immunoassay formats useful in low-resource settings. We introduce here new high-affinity chemical dye baits that bind the following well-characterized TB antigens and inflammatory markers: (i) LAM, (ii) early secretory antigenic target 6 (ESAT6) (*11*), (iii) culture filtrate protein 10 (CFP10) (*11*), and (iv) inflammatory cytokines nonspecifically associated with an active infection including interleukin-2 (IL-2), interferon-γ (IFN-γ), and tumor necrosis factor–α (TNFα) (*12*). For ESAT6, we examined the versatility of the cage nanotechnology as a new class of sandwich immunoassay using the chemical bait as one side of the sandwich.

## RESULTS

**Fig. 1:**
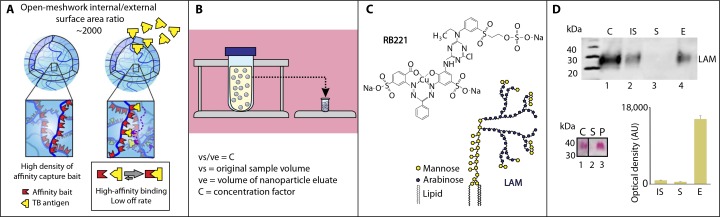
**Nanocages that were covalently functionalized with copper complex dye Reactive Blue 221 sequestered and concentrated lipoarabinomannan from urine**. (**A**) Schematic depicting high internal/external surface area ratio and binding capacity of nanocages. Affinity ligands covalently immobilized in the inner volume establish high-affinity noncovalent interaction with tuberculosis (TB) antigens. (**B**) Schematic showing the concentration factor given by the volumetric ratio between the initial urine volume and the final testing volume. Structures within the urine sample are nanocages. (**C**) Molecular structure of lipoarabinomannan (LAM) (right) and affinity probe Reactive Blue 221 (RB221) {cuprate(4-),[2-[[[[3-[[4-chloro-6-[ethyl[4-[[2-(sulfooxy)ethyl]sulfonyl]phenyl]amino]-1,3,5-triazin-2-yl]amino]-2-hydroxy-5- sulfophenyl]azo]phenylmethyl]azo]-4-sulfobenzoato(6-)]-,tetrahydrogen} (left). (**D**) Western blot, glycan staining, and image analysis of protein macroarray assay of LAM. C, LAM control (50 ng); IS, initial solution (50 ng of LAM spiked in 50 µl of human urine); S, supernatant; E, eluate from the nanocages; P, nanocages; AU, arbitrary units. Mean and SD, *n* = 3 replicates.

A recognized barrier to glycosciences and to the use of glycans as diagnostic biomarkers is the scarcity and suboptimal quality of available monoclonal antibodies (mAbs) directed against complex carbohydrates such as LAM (*13*). To address this problem, we introduce here a new class of chemical affinity bait, a copper complex reactive dye, Reactive Blue 221 {RB221; cuprate(4-),[2-[[[[3-[[4-chloro-6-[ethyl[4-[[2- (sulfooxy)ethyl]sulfonyl]phenyl]amino]-1,3,5-triazin-2-yl]amino]-2- hydroxy-5-sulfophenyl]azo]phenylmethyl]azo]-4-sulfobenzoato(6-)]-, tetrahydrogen} (Fig. 1). RB221 binds and sequesters carbohydrate glycan LAMantigenwith extremely high affinity (fig. S1) that is at least 100 times greater than any known lectin (fig. S2 and table S1). RB221 is immobilized in open-mesh hydrogel nanoparticle cages. When introduced into urine, the nanocages harvest LAM with high efficiency within minutes while simultaneously dissociating interfering substances in solution (fig. S3). Our affinity capture nanotechnology increases the sensitivity of LAM detection in urine by 100- to 1000-fold depending on the available volume of urine (Fig. 1, A and B) (*14*–*18*).

We applied this technology to study the concentration of LAM in the pretreatment urine of 48 HIV-negative patients with microbiologically confirmed active pulmonary TB from a Peruvian hospital (Tables 1 and 2). All patients were confirmed to have normal kidney function by in-hospital assessment including creatinine measurement and urinalysis. To determine whether urinary LAM concentrations reflect body disease burden, we compared the urine LAM concentrations with sputum TB organism counts, cough frequency, appetite, and change in bodyweight (*19*) in this cohort (Table 2) ofwell-characterized hospitalized patients using the widely accepted simplified nutritional appetite questionnaire (SNAQ) scoring system (*20*). Controls included age-matched healthy volunteers and diseased non-TB control patients who were hospitalized and ill with a variety of severe systemic, pulmonary, neurologic, and genitourinary tract diseases (table S2).

**Table 1:** **Demographic characteristics of study participants.** IQR, interquartile range.

	Median age, years (IQR)	Sex, M/F
TB patients (microbiological^ proven)	29 (22-37)	35/13
Healthy volunteers	26 (22-37)	24/15
Diseased TB-negative controls	32 (28-51)	9/5

**Table 2:** **Clinical characteristics of hospitalized patients (*n* = 48 microbiologically confirmed TB patients and *n* = 2 TB-negative patients).** Urine was collected from patients before therapy. SNAQ, simplified nutritional appetite questionnaire; MODS, microscopic observation broth-drug culture and susceptibility.

Microbiological data	
Auramine sputum smear microscopy result	
0 (%)	10
1 (%)	17
2 (%)	9
3 (%)	6
Paucibacillary (%)	7
MODS	
Positive	48
Negative	2
*Mycobacterium tuberculosis* isolate sensitive to	
Isoniazid	48
Rifampicin	48
Weight (kg)	52.9
SNAQ composite score	13.2
Self-reported symptoms	
Cough	
Yes (%)	38
No (%)	12
Hemoptysis	
Yes (%)	11
No (%)	39
Fever	
Yes (%)	32
No (%)	14
Fatigue	
Yes (%)	26
No (%)	24

### Copper complex dyes: High-yield LAM sequestration from urine

The carbohydrate structure of LAM(Fig. 1) (*3*) poses unsolved challenges in terms of identifying adequate probes for affinity isolation in urine in the presence of a vast excess of interfering urinary proteins and other biomolecules (*7*, *21*, *22*). For this study, 37 different dye chemistries (table S1) were screened to identify a molecular bait that would sequester LAM from urine with high affinity, deplete the supernatant, dissociate LAM-binding proteins, and permit a high-yield quantitative recovery (Fig. 1, C and D, and fig. S1). The dye chemistry panel was selected by inference from dyes known to be useful for tissue histology mucin staining or fluorescent staining ofmicroorganisms. Western blot analysis was conducted to screen dyes for their affinity to LAM. Two mAbs were tested and yielded highly specific bands for LAM with no detectable background in urine matrix (fig. S4). The specificity of the mAb clone CS-35was verified by antigen competition (fig. S5) (*23*).Dyes containing copper moieties for histologic staining are known to preferentially interact with glycans. Here, nanocages that were covalently functionalized with copper-containing dyes (Alcian blue pyridine variant and RB221) proved to be superior to other affinity probes, such as fluorescent brightener 28 (FB28), fast blue B, and safranin O (Fig. 1C and figs. S1, S2, and S6). Figure 1D documents the full depletion of LAM (100 ng/ml) spiked in human urine using the RB221 nanocages (Fig. 1D, lane S). Binding was independent of the pH of solution in the range 5 to 7 (fig. S7). Themolecularweight of the band by Western blot analysis and carbohydrate staining is the expected full size of LAM (~38,000) with no lower–molecular weight bands. After nanocage capture and elution, no differences were detected in the quantity, shape, or intensity of the LAM band captured in urine matrix as compared to LAM captured in phosphate-buffered saline (PBS) (Fig. 1D). Because PBS did not contain interfering substances, this verifies that the LAM was sequestered away from potential interfering urinary molecules including proteins, lipids, glycans, and cellular debris that could interfere with sequestration. On the basis of the intensity of the band compared to standards, the complete depletion of the supernatant at equilibrium, the yield and efficiency of capture and elution is greater than 95%. In human urine, on the basis of the bound versus free LAM at equilibrium, the capture affinity considerably exceeds *K*_d_ (dissociation constant) = 10^−9^ M (fig. S1).

Competition with 10% copper acetate in water or chelation by EDTA displaced LAM bound to RB221 (Fig. 1D, bar graph, and fig. S8), documenting the involvement of the copper moiety in the binding function. To further characterize the copper complex dye RB221 binding to the glycan, we used sodium *m*-periodate (NaIO_4_) oxidative degradation. As demonstrated by solid-phase immunoassay (fig. S9), NaIO_4_ at low concentrations and low pH extracted LAM from the RB221 cages, verifying that intactLAMdiolbonds are required for RB221 binding (fig. S9).

### Nanocage-based measurement of LAM in the pretreatment urine of patients with active pulmonary TB

**Fig. 2:**
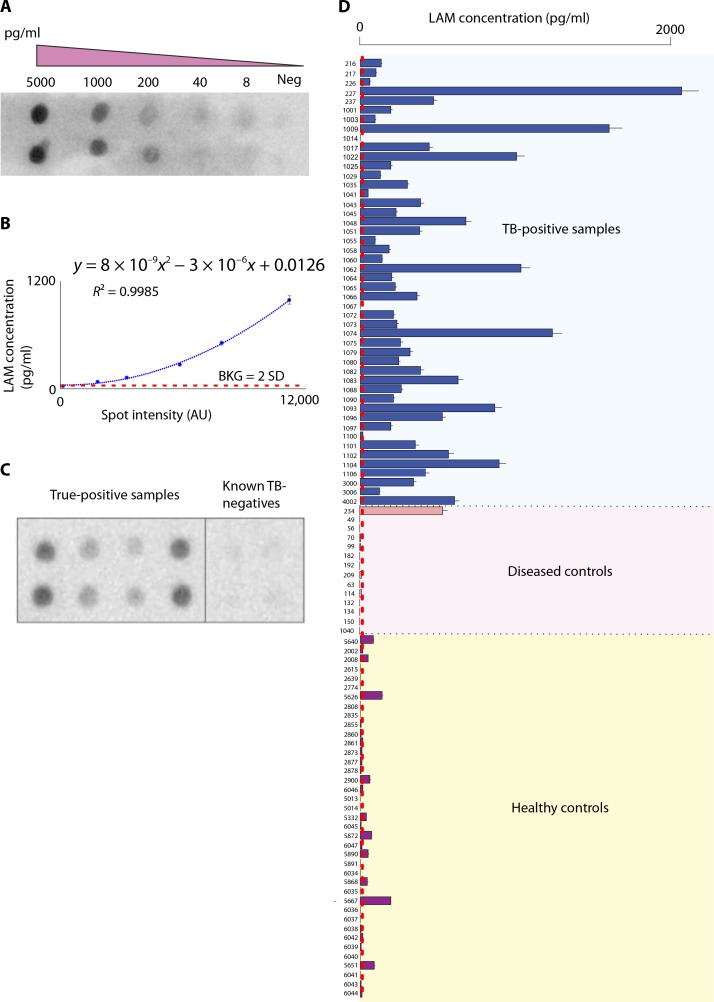
**LAMantigenwas detected in the urine of HIV-negative/TB-positive patients using RB221 nanocages for diseased and control patients listed in Table 1.** (**A**) Image of a quantitative immunomacroarray for LAMdetection, incorporating (**B**) a dilution curve in every membrane. Neg, negative; BKG, background. (**C**) Example immunomacroarray comparing urine samples from a set of true-positive and known TB-negative samples using nanocage preprocessing. (**D**) Bar plot of the intensities of LAM determined via immunomacroarray and ImageJ analysis from urine samples from healthy TB-negative, TB-negative diseased, and TB-positive patients shown in Table 1 (mean ± SD, *n* = 4 patient replicates).

Quantitation of LAM in human urine was performed after nanocage capture and elution using an immunomacroarray assay (*24*). The concentration of LAM in the reference calibrator was qualified by the anthrone colorimetric method in the linear portion of the assay (0.16 mg/ml; fig. S10). The concentration factor was 100-fold (Fig. 1). The immunomacroarray assay limits of detection and quantifications for 1ml of input urine were 14 and 15 pg/ml, respectively [background estimate, 547.32 arbitrary units (AU); SD, 22.6AU; lower limit of detection (LLD) = background + 2 * SD; and lower limit of quantification (LLQ) = background + 10 × SD; the polynomial equation *y* = 8 × 10^−9^*x*^2^ − 3 × 10^−6^*x* + 0.0126 (*R*^2^ = 0.9985) was used to estimate LAMconcentration (Fig. 2B)]. Unknowns were tested in an array with built-in negative controls and standards (Fig. 2). All samples and controls were identically processed through the nanocages.

### Verification of the assay format

A test set of 23 TB-positive patients and a verification set were analyzed (total *n* = 48 independent infected patient samples; Tables 1 and 2). The mean and SD of urinary LAM concentration in the test and verification sets were 700 ± 500 pg/ml and 410 ± 400 pg/ml, respectively. The two sets were statistically indistinguishable (Wilcoxon signed-rank test *P* = 0.07, *n* = 72). The patients were called positive if the LAM signal was 2 SD higher than the full process negative controls run simultaneously. LAM could not be measured in any of the TB-infected patient’s pretreatment urine without the nanocage concentration step.

### Urinary LAM: HIV-negative active TB-positive pretreatment patients discriminated from healthy and diseased controls

A total of 101 subjects qualified for the study (*n* = 48 microbiologically confirmed TB-positive patients, *n* = 14 diseased TB-negative patients, and *n* = 39 healthy volunteers). Informed consent was collected at the time of urine donation. The median age of the microbiologically confirmed TB patient was 29 years (interquartile range, 24 to 36), and 72% were males. The most commonly reported symptoms were cough (76%) and fever (64%). Demographic, clinical, and microbiological data are presented in Tables 1 and 2. Completed urine dipstick analysis was recorded (table S3). The full data results obtained with the immunomacroarray analysis described above are shown in Fig. 2D. For the truepositive patients (*n* = 48), only 2 had undetectable LAMconcentrations according to the criteria stated above. The controls included agematched healthy subjects and diseased non-TB patients who were ill with a variety of severe systemic, pulmonary, and urinary tract diseases. The diseases included pneumonia, lung cancer, pyelonephritis, genitourinary infection, sepsis, cryptosporidiosis, giardiasis, colon cancer with gastroenteritis, and liver failure (table S2).

**Fig. 3:**
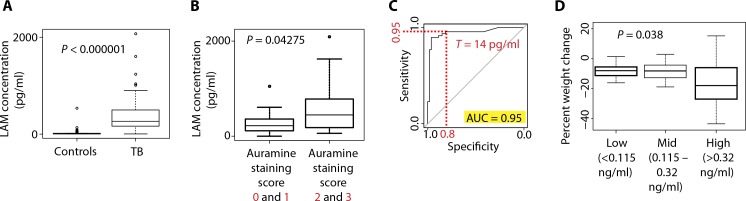
**Urinary LAM concentration predicted pulmonary TB and correlated to mycobacterial burden and weight loss.** (**A**) Box plot of the intensities of LAM in the urine of HIV-negative/TB-positive patients versus controls collected in endemic areas (Wilcoxon signed-rank test). (**B**) Box plot of the intensities of LAM in the urine of HIV-negative/TB-positive patients stratified on the basis of the auramine staining (low amount of microorganism, scores 0 and 1; high amount of microorganism, scores 2 and 3; Wilcoxon signed-rank test; *n* = 42). (**C**) Receiver operating characteristic analysis of the LAM intensity data. AUC, area under the curve. (**D**) Ordinal regression analysis shows statically significant correlation between the concentration of urinary LAM and the loss of body mass (*P* = 0.038, *n* = 37).

The difference in LAMconcentration between the cases and controls was highly significant [*P* < 1 × 10^−15^, *n* = 101, Wilcoxon signed-rank test; difference in location estimate, −247.318; 95% confidence interval (CI), −351.3 to 200.8; Fig. 3]. As shown in Fig. 3B, a significantly higher concentration of LAMwasmeasured in the urine of patients who had a higher score for sputum organism content (auramine score, *P* < 0.043, *n* = 42, Wilcoxon signed-rank test; difference in location estimate, −205.3; 95% CI, −452.0 to 5.1).

Sensitivity and specificity were evaluated by receiver operating characteristic (ROC) analysis, and the area under the curvewas calculated to be 0.95 (95% CI, 0.9005 to 0.9957; fig. S11) as an overallROCperformance [*n* = 48 cases, *n* = 53 controls; significance level, 0.05; power, 1 (*25*)]. At a threshold of 14 pg/ml, this ROC analysis yielded a sensitivity of 0.96 and a specificity of 0.81 for true-positive pulmonary TB patients in the present study set (positive predictive value, 0.82; negative predicted value, 0.95; power, 0.96) (Fig. 3C). By these criteria, the single false-positive urine in the diseased controlswas patient #234, whose urinalysis had +++ leukocyte esterase, +++ protein, +++ blood, and + bilirubin (table S3). These urinalysis values would meet the exclusion criteria for clinical urine diagnostic testing. Notably, eight of the nine culture-positive but smear-negative patients were positive for urinary LAM.

### Correlation of urinary LAM with clinical measures of disease burden and severity

Simple and multiple linear regression of covariates in Table 3 revealed that cough and appetite scoring compared to LAM urine concentrations were not individually significant by simple linear regression. However, when taken together, these two clinical measures were of significance and predictive of LAM urine concentrations (Table 3). Participants who reported a cough were likely to have an increased secretion of 269 pg/ml (10 to 528 pg/ml) of LAM (*P* = 0.042, *n* = 50). For appetite data, for each unit increase in SNAQ score (Table 4) (*20*), an increase of 54.1 pg/ml (4.76 to 103 pg/ml) ofLAMwas observed (*P* = 0.032, *n* = 37).

**Table 3:** **Simple and multiple linear regression analysis.** Analysis shows that cough and SNAQ scores (*20*, *36*), when combined, were significantly correlated to the concentration of urinary LAM. CI, confidence interval.

	Univariate (95% CI)	*P*	Multivariate (95% CI)	*P*
**Cough**	0.243 (-0.253 to 0.511)	0.075	0.269 (0.0100 to 0.528)	0.042 (*n* = 50)
**Eating habits**	0.049 (-0.001 to 0.100)	0.057	0.0541 (0.00476 to 0.103)	0.032 (*n* = 37)

**Table 4:** **SNAQ scoring (*****20*****,** ***36*****).**

Components of SNAQ score	LAM > 0.115 (*n* = 41)	LAM < 0.115 (*n* = 9)
My appetite is		
Very poor	2	0
Poor	14	4
Average	14	3
Good	9	1
Very good	2	1
When I eat		
I feel full after eating only a few mouthfuls	2	0
I feel full after eating about a third of a meal	2	0
I feel full after eating more than half a meal	10	3
I feel full after eating most of the meal	24	6
I hardly ever feel full	3	0
Food tastes		
Very bad	0	0
Bad	7	6
Average	24	1
Good	10	1
Very good	0	1
Normally I eat		
Less than one meal a day	0	0
One meal a day	3	0
Two meals a day	7	0
Three meals a day	26	6
More than three meals a day	5	3

Exploration of LAM as an ordinal variable revealed that the highest producers of LAM were those who had experienced the greatest change (loss) in bodymass as a proportion of their baseline mass (Fig. 3D). When patients were grouped into low-level LAM producers (115 pg/ml), mid-level LAM producers (115 to 320 pg/ml), and high-level LAM producers (320 pg/ml), high-level LAM producers lost, on average, 17%of their body mass as compared to patients in the low- and mid-level LAMproducing group who lost 8 and 9%of their body masses, respectively. Ordinal regression revealed a significant correlation of percent weight loss and LAM categorization (*P* = 0.038, *n* = 37; Table 5). This indicates that loss of weight in patients with high urinary LAM was consistent with a cachexia-like state characteristic of patients with advanced TB infection (*19*). These data are in keeping with the conclusion that the concentration of urinary LAMis a reflection of total mycobacterial body burden [auramine score (*26*)] and disease severity [cough and weight loss (*19*, *20*)] in patients with active pulmonary TB who are HIV-negative.

**Table 5:** **Ordinal regression analysis.** A significant correlation between the urinary LAM concentration and body mass change was observed.

	Low-level LAM (*n* = 6) (115 pg/ml)	Mid-level LAM (*n* = 15) (116-319 pg/ml)	High-level LAM (*n* = 16) (>320 pg/ml)
Mean percent -7.86 kg weight change	−7.86 kg	-8.63 kg	-16.97 kg
Odds ratio		0.933	
(95% CI)		(0.0873-0.996)	
*P*		0.038 (*n* = 37)	

### Extending the technology to other TB antigen markers and host-associated cytokines

**Fig. 4:**
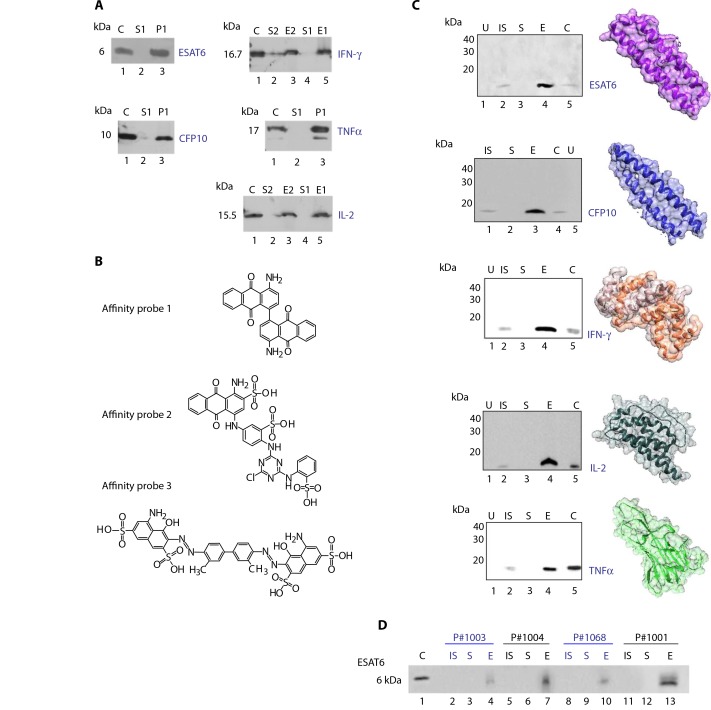
**Nanocages captured multiple TB-related analytes.** (**A**) SDS– polyacrylamide gel electrophoresis (PAGE) analysis; chemical bait incorporated in the nanocages (NP1, blue 3G-A; NP2, pigment red 177; NP3, disperse yellow 3). P, nanocage eluate. (**B**) Affinity probes (affinity probe 1, pigment red 177; affinity probe 2, blue 3G-A; affinity probe 3, trypan blue). (**C**) Nanocages effectively captured TB-related analytes from human urine (Western blot). U, negative control; C, recombinant protein (positive control, 75 ng). (**D**) Nanocage detection of TB antigen ESAT6 in the urine of untreated HIV-negative/TB-positive patients (Western blot).

Beyond LAM, additional low-abundance mycobacterial antigens that promise to offer important future diagnostic utility if they can be detected with adequate sensitivity in the complex matrix of urine have been characterized. We searched for bait chemistries that exhibited a high affinity for additional TB antigens (Fig. 4) such as ESAT6 and CFP10, which are secreted by replicating bacteria in addition to LAM shedding (*11*) during infection. We also explored bait chemistries for host immune response factors that, although not specific for TB diagnosis, may be involved in the cytokine cascade of TB infection [IL-2, TNFα, and IFN-γ (*27*)]. Results shown in Fig. 4 indicate that nanocages completely captured the target analytes, depleted the supernatant, and increased the effective concentration in the Western blot analysis. There was no cross-reactivity of the antibodies with the negative control human urine in the absence of target analytes. The analysis of urine samples from four untreated TB patients from the Peruvian cohort revealed that ESAT6 is detectable by Western blot analysis only when nanocages are used as a preprocessing step (Fig. 4D and Supplementary Materials and Methods). The four patients analyzed were characterized by microscopic observation broth-drug culture and susceptibility (MODS) TB culture (four of four are positive) and sputum smear (three of four are positive) (*28*). These data document that the nanocage technology is not limited to the LAM antigen and can be extended to other TB-related antigens to expand the detection panel and increase the accuracy of TB diagnosis.

### Nanocages can be magnetized

**Fig. 5:**
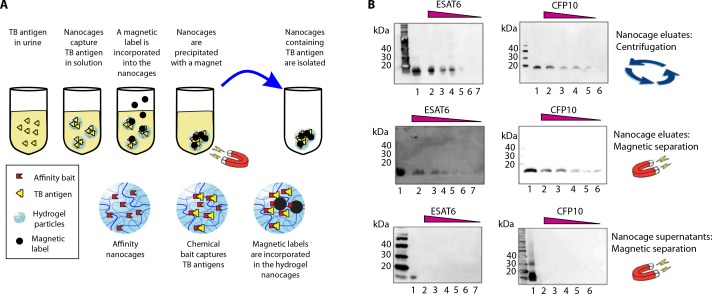
**Magnetic hydrogel nanocages.** (**A**) Schematic of magnetization. (**B**) Western blot analysis of ESAT6 and CFP10 expression in eluates of centrifugation-separated nanocages (top), in eluates of magnetic-separated nanocages (middle), and in supernatants after magnetic separation of nanocages from urine samples (bottom). Top and middle: Lane 1, positive control (recombinant protein; 10 ng); lanes 2 to 7, two to six eluates from nanocages incubated with 1 ml of urine containing ESAT6 (10, 5, 2.5, 1.2, 0.6, and 0.3 ng/ml) and CFP10 (10, 5, 2.5, 1.2, and 0.6 ng/ml). Bottom: Lane 1, positive control (recombinant protein; 10 ng); lanes 2 to 7, two to six supernatants after nanocage processing of 1 ml of urine containing ESAT6 (10, 5, 2.5, 1.2, 0.6, and 0.3 ng/ml) and CFP10 (10, 5, 2.5, 1.2, and 0.6 ng/ml).

Magnetization permits the creation of a urine collection device that achieves rapid separation of the particles from the urine in a self-contained vessel. To meet this goal, we incorporated a magnetic label (Fe_3_O_4_ functionalized with oleic acid; diameter, 100 nm) into the hydrogels (Fig. 5A and Supplementary Materials and Methods).As shown in Fig. 5B, magnetic separation is as efficient as centrifugation, if not superior, in separating the particles from urine and enabling the detection of ESAT6 and CFP10 at concentrations otherwise undetectable by Western blot analysis.

### Obviating the need for elution: Nanocages partially dissolve to display the captured analyte

**Fig. 6:**
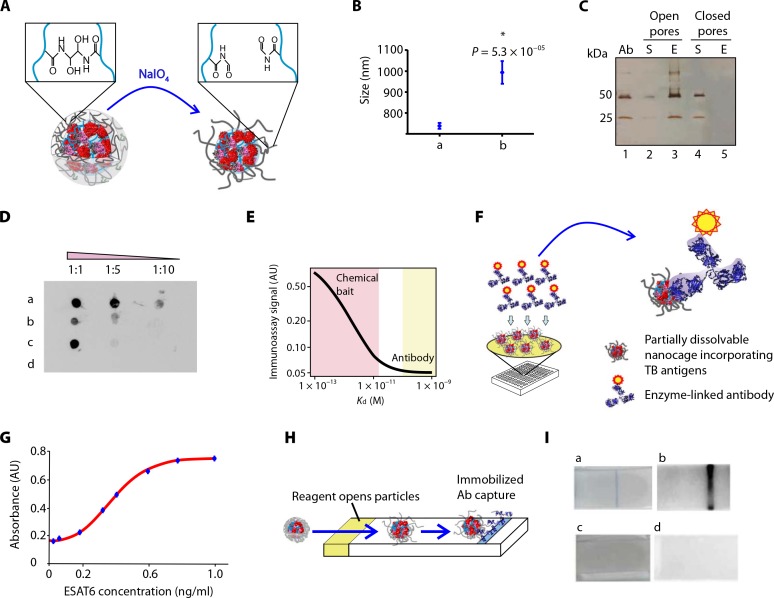
**Partially dissolvable nanocages captured antigen for antibody binding in a high-sensitivity sandwich immunoassay**. (**A**) Schematic demonstrating nanocage cross-link degradation in an oxidative environment. (**B**) Change in hydrodynamic diameter after nanocage oxidation [t test, *n* = 10; mean and SD of nanocage hydrodynamic diameter before (a) and after (**b**) oxidative degradation]. (**C**) SDS-PAGEanalysis comparing *N,N′*-(1,2-dihydroxyethylene) bisacrylamide (DHEA) cross-linked nanocages mixed with a solution of monoclonal antibody (Ab) (0.05 mg/ml) with pores open (lanes 2 and 3) and closed (lanes 4 and 5). (**D**) Immunomacroarray demonstrating that antigen bound to the chemical bait retains its capability to bind to the antibody. a, nanocages deposited on polyvinylidene difluoride (PVDF) membrane after incubation with urine containing ESAT6 (1 ml, 10 ng/ml) andDHEAcross-linkdegradation; b, nanocages deposited on PVDF membrane after incubation with urine containing ESAT6 (1 ml, 10 ng/ml) in the absence of DHEA cross-link degradation; c, ESAT6 deposited on PVDF membrane (starting amount, 1 ng); d, DHEA nanocages deposited on PVDF membrane after incubation with urine in the absence of ESAT6. (**E**) Plot of immunoassay signal intensity as a function of bait capture affinity. High-affinity chemical baits achieve >2 log increased sensitivity for antigen capture compared to conventional antibody, as mathematically demonstrated in Supplementary Materials and Methods. (**F**) Schematic depicting direct, nonelution sandwich immunoassay using partially degradable nanocages. Inset shows an enzyme-linked antibody interacting with TB antigens captured inside the nanocage. (**G**) Calibration curve of a direct nanocage immunoassay for ESAT6 showing linearity in the 1- to 0.03-ng range. (**H**) Schematic of a lateral flow immunoassay using one antibody. Nanocages capture and preserve antigen in solution, migrate through the filter membrane, and provide colorimetric detection. (**I**) Lateral flow immunoassay for ESAT6 detection in urine. Positive signal for 10 ng of ESAT6 in 10ml of human urine both visually (blue line, a) andwith chemiluminescence (black line, b). Negative control urine in the absence of ESAT6 yields no signal (c and d).

The effective pore size of the particles is a function of hydrogel polymer cross-links. Rendering the cross-links degradable provides a means to induce the nanocages to open and display the captured sequestered analyte (TB antigen) cargo. Partially degradable nanocages were created by incorporating a cleavable cross-linker (*N*,*N*′ -(1,2-dihydroxyethylene) bisacrylamide) under oxidizing conditions (Fig. 6, A to C, and Supplementary Materials and Methods) or *N*,*N*′-bis(acryloyl)cystamineunder reducing conditions (fig. S12). In this workflow, nanocages were mixed with urine containing the antigen of interest, and the solution-phase antigen was captured within the particles. The degradable cross-links were then cleaved, causing an effective increase in pore size and exposing the captured antigens in the internal volume. Antibodies were used to probe the exposed captured antigen directly within the cages for LAM (fig. S12) and ESAT6 (Fig. 6D).

### Single-antibody sandwich

This class of nanocages was then used as the basis for a single-antibody sandwich immunoassay forESAT6in a 96-well plate format.This format is completely distinct from conventional sandwich immunoassays because the capture antibody is replaced by the chemical bait and can yield improved analytical sensitivity (Fig. 6, E and F, and Supplementary Materials and Methods). The calibration curve for the assay is reported in Fig. 6G, indicating a high degree of linearity (*R*^2^ = 0.99) in the 1- to 0.03-ng range. This translates to a urine concentration sensitivity of 30 pg/ml for a 10-ml sample.

### Partially degradable nanocages: Lateral flow immunoassay

Degradable cages were successfully integrated in a lateral flow immunoassay (Fig. 6H and Supplementary Materials and Methods). A stripe of anti-ESAT6 antibody was printed on a glass fiber filter membrane. Nanocages were incubated with ESAT6-containing human urine, oxidized with NaIO_4_, and deposited on the glass fiber membrane. Cages migrated with a laminar flow and homogeneous front and were retained by the printed antibody, as shown in Fig. 6I. This class of cages permits nonelution immune-based detection of captured analytes directly sequestered and highly concentrated inside the cages (Fig. 6). The workflow is greatly simplified because the need for elution is obviated.

## DISCUSSION

### Shedding of urinary LAM in patients with active TB does not require HIV coinfection

This study indicates that it is possible to detect urinary LAM during active pulmonary TB, that detection does not require physiologic or immunologic consequences of HIV infection (*8*, *9*), and that detection is not limited to patients with TB colonization of the kidney (*10*). Instead, as others have suspected (*7*, *29*), the LAM antigen concentration in HIV-negative patients is below the level of sensitivity of previous immunoassays, and the urinary LAM may be obscured by interfering LAMbinding substances in the urine protein matrix (*7*). The high-affinity copper dye bait for LAM introduced in this study effectively sequesters LAM away from any potential urine-binding protein and concentrates the LAM by a large factor, depending on the input volume of the urine and the output total volume of the nanocages. At the same time, unwanted abundant urinary proteins are excluded. By comparison to published studies of LAM in the urine of HIV-positive, TB-coinfected patients (*5*, *6*), these data indicate that the concentration of LAM in the urine in active pulmonary TB, HIV-negative patients is 10- to 20- fold lower. This is in keeping with a greater than 10-fold lower total body burden of the TB pathogen in HIV-negative/TB-positive patients as assessed by polymerase chain reaction (*30*). HIV infection is known to increase the total body burden of the coinfecting pathogen (*30*), resulting in a higher number of organisms shedding their antigens into the urine.

### Urinary LAM shedding correlates with TB infection burden and severity

Concentrations of LAM in the pretreatment urine of infected patients were higher in patients with an increased body burden of infection as measured by TB organism count in the sputum and degree of patient weight loss. This is consistent with a cachexia-like state in patients with advanced TB infection (*19*). All of the patients in this study set were confirmed to have pulmonary TB by culture and were proven HIV-negative, and all had normal kidney function. Although subclinical TB infection of the kidneys and bladder may exist in some patients (*9*), it seems improbable that all patients in the cohort would have such a urinary tract infection. The molecular size of the LAM carbohydrate antigen is small enough to passively penetrate the normal glomerular filtration cutoff (*31*) to enter the urine. Thus, it is unlikely that a kidney infection is required for general urinary LAM shedding. Alternatively, our control patients who were TB-negative but harbored pyelonephritis and other genitourinary infections were negative in our LAM immunoassay.

The present studywas performed using pretreatment urine. It will be important to compare urinary LAMin patients before and after therapy to evaluate potential therapy-induced reduction or elevation in LAM antigen shedding. The concentration factor afforded by the nanocage technology readily measured urinary LAM in >96% of the urine of true-positive patients (2 SD above background), compared to healthy negatives and diseased controls. As shown in Fig. 3, the Denkinger *et al*. (*2*) requirements of 95% sensitivity and >80% specificity for a useful screening tool were achieved in this study set. Nevertheless, this must be extended to a larger cohort of patients for further validation using one or more of the rapid formats described in Fig. 6.

Note that eight of the nine patients whowere TB smear-negative and culture-positive were positive for urinary LAM. Sputum smear microscopy for acid-fast bacilli has a sensitivity of 50 to 60%; this sensitivity can be even lower in resource-constrained settings because of the requirement of a trained microscopist (*26*). Thirteen to 17% of newly transmitted TB cases are attributed to patients who are smear-negative but ultimately culture-positive (*32*). These data support the conclusion that testing methods with adequate sensitivity, superseding the drawbacks of smear microscopy, can now be used to routinely measure LAMin the urine of patients with active pulmonary TB. This has broad implications for pulmonary TB screening, transmission control, and treatment management.

### Chemical affinity baits and nanocage chemistry offers many options for TB antigen testing

Enhanced sensitivity for low-abundance analytes is attained by affinity separation and concentration of the diagnostic analytes from a large volume of input sample.On the basis of a urine volume of 40ml (one urine collection cup), in future applications, the concentration factor would be 1000, because all of the analyte in the urine sample is concentrated into 40 ml. We screened a large series of previously uncharacterized dyes to achieve the highest affinity for binding TB antigens (Figs. 1 and 4 and table S1). The specificity of the nanocage-enhanced assay is derived not from the high-affinity bait but from the downstream analyte detection system (*14*–*18*). Specifically, the copper dye chemistry used in the assay was selected on the basis of its unusually high affinity for LAM that far surpasses other dyes, including the FB28 dye commonly used to stain bacterial surface glycans (*33*), and greatly exceeds known affinities of lectins (*34*). In the case of LAM, the specificity of the assay is provided by a mAb that recognizes an epitope on the branched carbohydrate LAM moiety (*23*).

### Extension of the technology to measure other TB analytes

The following are the limitations of this study: (i) the relatively small patient and diseased control sample size, (ii) the small volume of pretreatment urine analyzed (1 ml) (although we demonstrated lack of cross-reactivity for *Neisseriameningitidis* and *Streptococcus pneumoniae*, it would be valuable to verify specificity against polysaccharides from other pathogenic bacteria; if a larger volume of urine is analyzed, then a proportionally higher analytical sensitivity can be reached), and (iii) the extra step required to elute the LAM antigen cargo from the nanocage. Dissolvable cages can overcome the need to elute the captured antigens. The utility of the technology can be expanded to detect other major diagnostic analytes of interest for TB screening: ESAT6 and CFP10, as well as IL-2, IFN- γ, and TNFα. Host cytokines IL-2, IFN-γ, and TNFα are elevated during infection and have been suggested as markers of active infection, although not specific for TB (*12*). All of thesemarkers have been difficult or impossible to measure in urine because of their low concentration. Using the nanocage technology, we have verified the presence of ESAT6 in the urine of a pilot set of four HIV-negative/ TB-positive patients. In the past, it has been impossible to longitudinally track patientswith latent TBwho are switching to active disease. It is conceivable that measuring a panel of host and pathogen analytes in the urine will reveal a signature of the switch from latent to active disease. In the past, noninvasive, low-cost, quantitative urine tests were not sensitive enough to detect active TB in HIV-negative patients and gauge the severity of the disease, particularly in low-resource settings. These questions can now be addressed with the analytical methods introduced here.

## MATERIALS AND METHODS

### Study design

The goal of the study was to develop a noninvasive urinary test for active pulmonary TB that achieved high sensitivity and specificity. An analyteharvesting technology was created to capture and concentrate the TBassociated antigen LAM. The first phase of this study was a clinical testing phase. The technology was used to quantitatively measure urinary LAM of TB patients and correlate LAM concentrations with TB disease severity. Sensitivity and specificity comparing true positives (*n* = 48) with healthy (*n* = 39) and diseased (*n* = 14) controls were assessed with ROC curves and Wilcoxon tests. The second phase of this study documented the versatility of the technology for additional TB-related analytes ESAT6 and CFP10 and relevant host cytokines. Sandwich and lateral flow immunoassay feasibility for testing clinical specimens was documented for ESAT6.

### Patient study cohorts

Urine samples were collected from hospitalized patients in Peru. Diagnosis of active pulmonary TB was verified microbiologically by auramine staining for acid-fast bacilli in sputum and MODS assay (*28*). The relative intensity of auramine staining for acid-fast organisms was scored from 0 to 3, with 3 being the highest (*26*). Specimens were collected under informed consent; the study received institutional review board approval at the Universidad Peruana Cayetano Heredia (Lima, Peru) and Johns Hopkins Bloomberg School of Public Health (Baltimore,MD). Clinical and demographic data included age, sex, previous TB diagnosis, weight, appetite, self-reported symptoms (including cough, hemoptysis, fever, and fatigue), and average number of coughs in the previous 24 hours. Appetite was assessed using the SNAQ (*20*), which has been validated to assess appetite and weight loss in ambulatory patients in a range of settings (*20*). Urine samples were immediately centrifuged at 3000 relative centrifugal force (rcf) for 10 min, and the supernatantswere stored in liquid nitrogen or −80°C until use.Urine specimens were collected from 14 hospitalized diseased controls and 39 healthy volunteers (Table 1). Negative diseased controls were collected from the same geographical area. Patient urine samples were qualified before the analysis by urinary dipstick testing (Multistix GP, Siemens) for hematuria, proteinuria, cystitis, and specific gravity analysis for each case. Urinary nitrites were scored in the urine because they can be a product of oxidation of nitrates in the TB-infected tissue microenvironment (*35*). Here, nitrites were not a parameter for excluding patients.

### Nanocage production

Hydrogel cages were produced by precipitation polymerization (*14*); high-affinity dye molecules were covalently bound to the hydrogel cages as reported by Tamburro *et al*. (*14*). RB221 (OrganicDyes and Pigments), trypan blue (Sigma-Aldrich), remazol brilliant blueR(RBB; Sigma-Aldrich), disperse yellow 3 (DY3; Fluka), FB28 (Sigma-Aldrich), and pigment red 177 (PR177; International LaboratoryUSA)were verified by thin-layer chromatography. Comonomers listed in Table 6 were dissolved in Milli-Qwater and filtered (0.45mm, Millipore). The solution was purgedwith nitrogen at room temperature at medium stirring for 1 hour and then heated to 50°C (for 1:NBaAl) or 70°C (for 2:NBiAc, 3:NBiDAc, and 4:NBiNh). Potassiumpersulfate was added to initiate the polymerization under nitrogen for 6 hours. The resulting cages were washed five times (54,400 rcf for 50 min at 25°C) and then resuspended in Milli-Q water.

**Table 6:** **Hydrogel nanocage synthesis: Quantity (in millimoles) of comonomers and total volume of reaction.** NIPAm, *N*-isopropylacrylamide; BIS, *N,N'*-methylenebisacrylamide; DHEA, *N,N'*-(1,2-dihydroxyethylene) bisacrylamide; AAc, acrylic acid; KPS, potassium persulfate, TEMED, *N,N,NVV'*- tetramethylethylenediamine; BAC, *N,N'*-bis(acryloyl)cystamine; AA, allylamine; NHA, *N*-(hydroxymethyl)acrylamide.

	NIPAm	BIS	DHEA	BAC	AAc	AA	NHA	TEMED	KPS	Volume
1:NBaAl	39	—	—	0.9	—	4.5	—	0.17	0.18	150
2:NBiAc	42	2.6	—	—	7.3	—	—	—	1.02	500
3:NBiDAc	6.16	0.64	0.64	—	0.56	—	—	—	0.17	70
4:NBiNh	42	2.6	—	—	—	—	7.3	—	1.02	500

RB221 (Table 7) was coupled to the cages as follows: 0.3 g of RB221 powder wasmixed to a solution obtained by adding 0.66 g of Na_2_CO_3_ to 50 ml of deionized (DI) water and stirring at a medium rate until it is completely dissolved. The solution was filtered (0.45-µm pore size). Fifty milliliters of 1:NBaAl cage suspension obtained as described above was added and allowed to incubate overnight at room temperature. RB221 coupled cages were washed five times (54,400 rcf for 50min at 25°C) and resuspended in 50 ml of DI water.

**Table 7:** **Dye coupling to cages.** RB221, reactive blue 221; RBB, remazol brilliant blue R; DY3, disperse yellow 3; PR117, pigment red 177; FB28, fluorescent brightener 28.

RB221	Trypan blue (*17*)		RBB (*14*)	DY3 (*14*)	PR177 (*14*)	FB28
1:NBaAl	2:NBiAc,	3:NBiDAc	2:NBiAc	2:NBiAc	2:NBiAc	4:NBiNh

FB28 (Table 7) was coupled to the cages as follows: The pH of 100-ml 4:NBiNh cages was adjusted to 3 by adding 1 M HCl. The nanocage solution was heated to 70°C. FB28 (0.54 g) was dissolved in 10 ml of water, added, and kept at 70°C under stirring for 6 hours. FB28-coupled cages were washed and resuspended in 100 µl of DI water. Trypan blue, RBB, DY3, and PR177 were covalently incorporated in the cages as described in Supplementary Materials and Methods and by Tamburro *et al*. (*14*). Incorporation of the dye into the internal volume of the cage was verified by differential capture yield in cages where pore size was modulated by selective chemical degradation of the cross-links (fig. S12 and Supplementary Materials and Methods).

### Nanocage enrichment and detection of TB antigens

Immunomacroarray analysis was conducted as follows. Purified LAM from the *Mycobacterium tuberculosis* strain H37Rv was obtained from BEI Resources (catalog #NR-14848). Anti-LAM antibody was obtained from BEI Resources (NR-13811 LAMmAb clone CS-35).One milliliter of human urine was incubated with 100 µl of nanocage suspension (5 mg/ml, dry weight). Cages were separated from urine by centrifugation, washed with DI water,mixed with 10 µl of Novex 2×Tris-Glycine SDS Sample Buffer (Thermo Fisher Scientific) containing 10% (v/v) 2-mercaptoethanol, and incubated at 100°C for 2min. The cage suspension was centrifuged (16,100 rcf for 10min at 25°C), and the supernatant was saved and subjected to detergent removal (HiPPR Detergent Removal Resin Column Kit, Thermo Fisher Scientific) according to the vendor’s instruction and using 100 µl of bead suspension. Aliquots (4 µl) of the resulting purified elution weremanually spotted on polyvinylidene difluoride membranes previously activated with methanol and rinsed with DI water. Membranes were allowed to dry at room temperature and then stained using anti-LAM CS-35, horseradish peroxidase–labeled anti-mouse antibody, and enhanced chemiluminescence system (Super- Signal West Dura, Thermo Fisher Scientific).

### Statistical analysis

Statistical differences between TB-positive and TB-negative patients and low and high microorganism burden were assessed by two-sided Wilcoxon rank sum test with an a level of 0.05. Regression analysis was performed with STATA 13. The relationship between covariates and outcome was explored using linear, logistic, and ordinal regression. Forward and backward stepwise regression was used to optimize covariate selection. The covariates of interest were indicators of socioeconomic status, clinical symptoms (such as cough, fever, and weight loss), and indicators of appetite. Assessment of appetite was performed using the SNAQ (*20*). Survey questions included how the appetite of patients had been leading up to their hospital admission, how much food they liked to consume in a meal, how they felt their food tasted, and how many times a day they were eating. Ratings were completed on a five-point scale, and a higher score equals higher eating frequency. Patients were asked about their normal weight during the initial study survey and weighed upon admission to the hospital. Percent weight change is used as a covariate. Primary outcome was detection of LAM in patient urine. LAM was considered as a linear, binary (urinary LAM cut point, 115 pg/ml), and ordinal outcome (urinary LAM < 115 pg/ml, 115 pg/ml < urinary LAM < 320 pg/ml, and urinary LAM > 320 pg/ml). Statistical significance of regression coefficients was assessed by two-sided t tests and z test with an a level of 0.05. Statistical difference in the hydrodynamic diameter between partially degraded and intact nanocages was assessed by two-sided t tests with an a level of 0.05. Densitometry analysis of Western blot and immunomacroarray signals was conducted using ImageJ software. Box plot, ROC analysis, and power calculations (*25*) were conducted using R statistical software (www.r-project.org/).

## SUPPLEMENTARY MATERIALS

www.sciencetranslationalmedicine.org/cgi/content/full/9/420/eaal2807/DC1

Materials and Methods

Fig. S1. The Kd affinity between RB221 and LAM exceeds that of FB28.

Fig. S2. Copper dyes outperform copper free dyes such as fast blue B and safranin O.

Fig. S3. Nanocages dissociate biomarker from interfering substances, in silico mathematical modeling.

Fig. S4. CS-35 mAb is specific for LAM diluted in human urine, batch verification.

Fig. S5. Competition assay confirmed the specificity of CS-35 mAb.

Fig. S6. Coupling chemistry to covalently incorporate the FB28 dye in the inner volume of the nanocages.

Fig. S7. LAM binding to RB221 and depletion from supernatant are independent of pH in a 5 to 7 range.

Fig. S8. RB221 binding to LAM is hindered by the presence of a copper-chelating agent (EDTA).

Fig. S9. RB221-LAM interaction requires intact diol moieties of LAM as proven by NaIO4 oxidation.

Fig. S10. Carbohydrate concentration in the LAM reference standard (0.160 mg/ml) was quantified by a linear colorimetric assay.

Fig. S11. Plot of the 95% CI of the sensitivity and specificity of the ROC analysis reported in

Fig. 3C.

Fig. S12. The RB221 dye is immobilized in the inner volume of the cages and is available for high–molecular weight ligand binding after cross-link degradation and consequent increase of the effective pore size.

Fig. S13. CS-35 anti-LAM mAb does not cross-react with purified polysaccharides from N. meningitidis and S. pneumoniae.

Fig. S14. Nanocage capturing followed by CS-35 antibody detection is specific for LAM and does not cross-react with M. tuberculosis lipomannan and arabinogalactan.

Table S1. Nanocage bait chemistries screened to capture and enrich LAM from human urine.

Table S2. Medical characteristics of diseased TB-negative controls.

Table S3. Urinalysis results for all study participants.

## Supplementary Material

Urine lipoarabinomannan glycan in HIV-negative patients with pulmonary tuberculosis correlates with disease severity
