# Supplementary material for: Urine lipoarabinomannan glycan in HIV-negative patients with pulmonary tuberculosis correlates with disease severity
Source: Sci Transl Med. 2017 Dec 13;9(420):eaal2807. doi: 10.1126/scitranslmed.aal2807 (PMC6037412; doi:10.1126/scitranslmed.aal2807)
Supplement: Urine lipoarabinomannan glycan in HIV-negative patients with pulmonary tuberculosis correlates with disease severity [file STM-09-eaal2807-s001.pdf]

Science Translational Medicine
AAAS

www.sciencetranslationalmedicine.org/cgi/content/full/9/420/eaal2807/DC1

Supplementary Materials for

Urine lipoarabinomannan glycan in HIV-negative patients with pulmonary tuberculosis correlates with disease severity

Luisa Paris, Ruben Magni, Fatima Zaidi, Robyn Araujo, Neal Saini, Michael Harpole, Jorge Coronel, Daniela E. Kirwan, Hannah Steinberg, Robert H. Gilman, Emanuel F. Petricoin, Roberto Nisini, Alessandra Luchini,* Lance Liotta

*Corresponding author. Email: aluchini@gmu.edu

Published 13 December 2017, *Sci. Transl. Med.* **9**, eaal2807 (2017)
DOI: 10.1126/scitranslmed.aal2807

This PDF file includes:

Materials and Methods
Fig. S1. The K_d affinity between RB221 and LAM exceeds that of FB28.
Fig. S2. Copper dyes outperform copper free dyes such as fast blue B and safranin O.
Fig. S3. Nanocages dissociate biomarker from interfering substances, in silico mathematical modeling.
Fig. S4. CS-35 mAb is specific for LAM diluted in human urine, batch verification.
Fig. S5. Competition assay confirmed the specificity of CS-35 mAb.
Fig. S6. Coupling chemistry to covalently incorporate the FB28 dye in the inner volume of the nanocages.
Fig. S7. LAM binding to RB221 and depletion from supernatant are independent of pH in a 5 to 7 range.
Fig. S8. RB221 binding to LAM is hindered by the presence of a copper-chelating agent (EDTA).
Fig. S9. RB221-LAM interaction requires intact diol moieties of LAM as proven by $NaIO_4$ oxidation.
Fig. S10. Carbohydrate concentration in the LAM reference standard (0.160 mg/ml) was quantified by a linear colorimetric assay.
Fig. S11. Plot of the 95% CI of the sensitivity and specificity of the ROC analysis reported in Fig. 3C.

Fig. S12. The RB221 dye is immobilized in the inner volume of the cages and is available for high–molecular weight ligand binding after cross-link degradation and consequent increase of the effective pore size.
Fig. S13. CS-35 anti-LAM mAb does not cross-react with purified polysaccharides from *N. meningitidis* and *S. pneumoniae*.
Fig. S14. Nanocage capturing followed by CS-35 antibody detection is specific for LAM and does not cross-react with *M. tuberculosis* lipomannan and arabinogalactan.
Table S1. Nanocage bait chemistries screened to capture and enrich LAM from human urine.
Table S2. Medical characteristics of diseased TB-negative controls.
Table S3. Urinalysis results for all study participants.

Materials and Methods

Theoretical justification for affinity capture and sensitivity

The signal response of an immunoassay can be expressed via Four Parameter Logistic Model. According to the law of mass action, when the binding reaction between an affinity probe A and the antigen C $A + C = AC$ reaches the equilibrium, the ratio between the concentration of product AC and the reactants C and A is constant and can be expressed as $\frac{[A][C]}{[AC]} = K_D$. If we combine the logistic model and the law of mass action, we obtain:

$$R = T + \frac{B-T}{1+\left(\frac{[C]}{EC50}\left(1+\frac{[A]}{K_D}\right)\right)^S} \qquad \text{Eq.S1}$$

Where R is the expected signal response of the immunoassay, T is the top asymptote of the antibody dose response curve, B is the bottom asymptote of the antibody dose response curve, EC50 is the concentration of antigen at which the signal response of the immunoassay is halfway between T and B, and S is the slope of the of the antibody dose response curve at the mid asymptote point. This mathematical equation reveals that lower KD, equivalent to higher affinity of the probe towards the target analyte, yields higher signal intensity (Fig. 6E). This provides theoretical justification why the high affinity chemical baits increase the sensitivity of the assay compared to the conventional sandwich immunoassay.

Theoretical justification for high affinity sequestration of TB antigens in a urine matrix.

Nanocages sequester low molecular weight or low abundance TB antigens (biomarkers) from complex biological matrices, even though the biomarker is complexed with high abundance proteins. The capturing process can be demonstrated by the following simple mathematical model. At *t=0,* a harvesting capture molecule is introduced to the system, at which time an equilibrium is assumed to exist between the free (uncomplexed) biomarker, *b*, the 'natural'

capture carrier protein, C_N, and the biomarker-carrier protein complex, bC_N. In the absence of a harvesting carrier molecule, the reaction scheme is as depicted below,

$$b + C_N \underset{k_{-N}}{\overset{k_N}{\rightleftharpoons}} bC_N$$

Eq. S2

where k_N and k_{-N} are the forward and reverse rate constants for the reaction, respectively. Here it is assumed that the carrier protein exists in such vast excess over the biomarker that the free carrier protein concentration, $[C_N]$, may be considered a constant ($[C_N] >> [bC_N]$ $\forall$ $[bC_N]$ $\Rightarrow$ $[C_N] \approx [C_N]$), and may be absorbed into the forward rate constant. Thus, the equilibrium ratio will be given by

$$\frac{[bC_N]}{[b]} = \frac{k_N}{k_{-N}} = K_N,$$

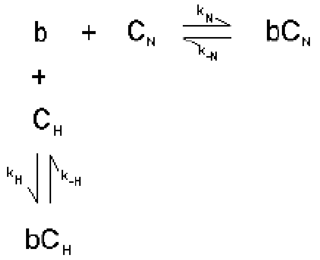

Eq. S3

where K_N is the affinity constant for the biomarker and its natural carrier protein. Note that $k_N >> k_{-N}$ or $K_N >> 1$ since the relatively large free carrier protein concentration typically amplifies the forward reaction rate by several orders of magnitude. When the harvesting capture cage is introduced, on the other hand, the reaction scheme is as depicted in Equation S4,

$$bH \underset{k_H}{\overset{k_{-H}}{\rightleftarrows}} H + b^* \overset{k_D}{\leftarrow} b + C \underset{k_{-c}}{\overset{k_c}{\rightleftarrows}} bC$$

Eq. S4

$$\frac{d[b]}{dt} = k_{-C}[bC] - k_C[b] - b_{in}$$
$$\frac{d[b^*]}{dt} = b_{in} + k_{-H}[bH] - k_H[b^*]$$
$$\frac{d[bC]}{dt} = k_C[b] - k_{-C}[bC]$$
$$\frac{d[bH]}{dt} = k_H[b^*] - k_{-H}[bH]$$
$$b_{in} = f(r,p)([b]-[b^*]) = k_d.r.p.([b]-[b^*])$$

Eq. S5

where k_H and k_{-H} are the forward and reverse rate constants, respectively, for the reaction between the biomarker and the harvesting capture cage, C_H, producing the complex bC_H. Note that the rate constants k_H and k_{-H} account for the rate of biomarker transit through the cage pores as well as the affinity of the biomarker for the encapsulated bait molecule. In the present case since the nanocages are buoyant open mesh, b = b* and diffusion effects are negligible.

Here again, the harvesting cages are present in such vast excess over the low abundance biomarker that their (very large) concentration may be absorbed into the forward rate constant, k_H, so that $k_H >> k_{-H}$ The introduced harvesting cages have a much greater affinity for the small biomarkers, in addition to existing at a higher concentration compared to the natural carrier proteins, so that $k_H >> k_N$. For this reason, an association between the biomarker and the harvesting cage is markedly preferred over the complex with the natural carrier protein. Since the harvesting cage concentration itself contributes to the forward rate constant, k_H, this bias towards the harvesting cage will be enhanced further as the concentration of harvesting cages in the mixture is increased. The temporal variations of the free and complexed forms of the biomarker are now described by the suite of Equations (2) through (4) below, subject to the specified initial conditions and parameter set.

Parameters and Initial Conditions for Equations (S6)-(S8)

$\frac{d[b]}{dt} = -k_N[b] + k_{-N}[bC_N] - k_H[b] + k_{-H}[bC_H]$ Eq. S6

$\frac{d[bC_N]}{dt} = k_N[b] - k_{-N}[bC_N]$ Eq. S7

$\frac{d[bC_H]}{dt} = k_H[b] - k_{-H}[bC_H]$ Eq. S8

$k_N = 0.1$; $k_H = 1$; $k_{-N} = k_{-H} = 0.01$; $K_N = 10$; $[bC_N](0) = 1$; $[b](0) = \frac{[bC_N](0)}{K_N} = 0.1$; $[bC_H](0) = 0$.

Note that this parameter set has been chosen so that $k_N >> k_{-N}$ ($K_N >> 1$), $k_H >> k_{-H}$ ($K_H >> 1$) and $k_H >> k_N$. In other words, biomarkers markedly prefer complexed forms over free forms, and the formation of a complex with the harvesting molecule is markedly preferred over a complex with the natural carrier protein. The solutions to these equations are depicted in **Figure S1**, for a harvesting cage with a biomarker affinity that is ten times greater than that of the natural carrier protein, $k_H/k_N=10$

The mechanism underlying these model solutions may be explained as follows: When the harvesting cages are first introduced to the mixture, the bound and free-phase biomarkers are initially in the ratio determined by Equation S2, $[bC_N]=K_N[b]$. The harvesting cages immediately begin to sequester the free-phase biomarker, attempting to establish the corresponding ratio $[bC_H]=K_H[b]$ between the complexed and uncomplexed biomarker forms. This removal of free-phase biomarker from the mixture perturbs the equilibrium ratio for the natural urinary carrier protein, so that $[bC_N]<K_N[b]$, which in turn generates a driving force for the transfer of biomarker from the natural carrier protein to the free-phase form. This transfer sponsors the

continued binding of free-phase biomarker to the harvesting cages. Biomarker transfer from the natural carrier protein to the harvesting cage via the free-phase form will continue in this way until an overall equilibrium is reached, with $[bC_H] = K_H[b]$ and $[bC_N]=K_N[b]$. The proportion of biomarker in complexed association with the harvesting cage will then be $K_H/(1+K_N+K_H) \approx K_H/(K_N+K_H)$. Thus, the greater the biomarker affinity for the harvesting cage, K_H, in comparison with its affinity for the natural urinary carrier protein, K_N, the closer this ratio will be to unity (all biomarker in complexed association with the harvesting cage.)

Equilibrium constant of LAM binding to dyes in the nanocages.

The following simple model was used to describe the interaction between LAM and the dyes incorporated in the nanocages (fig. S1):

$$L + B \underset{kd}{\overset{ka}{\rightleftarrows}} LB \qquad \text{Eq. S9}$$

Where L = LAM in solution, B = dye affinity bait, LB = LAM associated to the dye in the cages, kd =dissociation constant, ka= association constant. Integration of the following differential equations was used to fit the association and dissociation kinetics, respectively:

d[LB]/dt = ka[L][B] – kd[LB]

d[LB]/dt = -[LB].

The parameters were used:

$[L]_0$ = 5.4e-08 M; [B] = const=1.2e-4 M for reactive blue 221

$[L]_0$ = 2.5e-08 M; [B] = const = 1.2e-3 M for fluorescent brightener 28.

Production of nanocages functionalized with RB221, trypan blue, RBB, DY3, PR177, Fast Blue B and Safranin O.

1:NBaAl. N-Isopropylacrylamide (NIPAm, 4.5 g, 39 mmol), N, N'- bis(acryloyl)cystamine (BAC 236 mg, 0.9 mmol) and allylamine (AA, 338 µl, 4.5 mmol) were dissolved in 150 mL of water, filtered, and transferred into a three-neck round bottom flask using a 0.45 µm nitrocellulose membrane disk filter. The system was purged with nitrogen for 30 minutes at room temperature and medium stirring rate and then heated to 50°C. N,N,N',N'-tetramethylethylenediamine (TEMED, Thermo Fisher, 19.4 mg, 0.17 mmol) was added to the solution and after 10 minutes potassium persulfate (50 mg, 0.18 mmol) was added to initiate the polymerization. The system was held at 50°C under nitrogen for 4 hours. Nanocages were then washed five times with water in order to eliminate the unreacted reagents (19,000 rpm, 50 min, 25 °C).

2:NBiAc. NIPAm (4.750 g, 42 mmol), N,N'-methylene bisacrylamide (BIS, 0.400 g, 2.6 mmol), and acrylic acid (AAc, Sigma-Aldrich, 0.525 g, 7.3 mmol) were dissolved in 500 ml of MilliQ water and filtered using nitrocellulose membrane (0.45 µm, Millipore, MA-USA). The solution was purged with nitrogen at room temperature at medium stirring rate for 1 h, and then heated to 70 °C. Potassium persulfate (KPS, Sigma-Aldrich, 0.276 g, 1.02 mmol) was added to initiate the polymerization. The reaction was maintained at 70 °C under nitrogen for 6 h. The resulting cages were washed five times by centrifugation (19,000 rpm, 50 min, 25 °C) to eliminate the unreacted monomer and then resuspended in 500 mL of MilliQ water.

3:NBiDAc NIPAm (697 mg, 6.16 mmol) *N,N'*-(1,2-Dihydroxyethylene)bis-acrylamide (DHEA, 128mg, 0.64 mmol), BIS (99mg, 0.64 mmol) and AAc (38 µL, 0.56 m mol) were dissolved in 65 mL of H2O, filtered using a nitrocellulose membrane disk filter (0.45 µm pore size, Millipore), and transferred in a three-neck round-bottom flask. The solution was purged with nitrogen for 1 h at room temperature, at medium stirring rate, and then heated to 70 °C. Potassium persulfate

(KPS, Sigma-Aldrich, 46 mg, 0.17 mmol) was dissolved in 5 mL of H2O and was added to the solution to initiate the polymerization. The reaction was maintained at 70 °C under nitrogen for 4 h. Nanocages were washed five times by centrifugation (19,000 rpm, 50 min, 25 °C) to eliminate the unreacted monomer and then re-suspended in 70 mL of H2O.

Reactive Blue 221 (RB221) 300 mg of RB221 powder were mixed to a solution obtained by adding 0.66 g of Na2CO3 to 50 ml of DI water and let it stir at medium rate for few minutes until completely dissolved. RB 221 solution was filtered using a nitrocellulose membrane disk filter (0.45 μm pore size). 50 mL of 1:NBaAl cages were added and let incubate overnight at room temperature. Cages were washed five times by centrifugation (19,000 rpm, 50 min, 25 °C) to eliminate the unreacted dye and then re-suspended in 50 mL of DI water.

Trypan Blue (TB), Remazol Brilliant Blue R (RBB) and Disperse Yellow 3 (DY3). 10 mL of 2:NBiAc or 3:NBiDAc cages (acrylic acid moles = 0.0000121) was activated in a solution containing 1 mL of 1% w/v SDS (Sigma), 0.2 M NaH_2PO_4 (Sigma, pH = 5), 0.0042 mol of N-(3 Dimethylaminopropyl) N' ethyl carbodiimide hydrochloride (EDC; Fluka Analitical) and 0.0052 mol of solid N-Hydroxy succinimide (NHS; Sigma-Aldrich) for 15 minutes. The dye (RBB, TB, and DY3, molar ratio of dye /acrylic acid 10:1) was dissolved in 180 mL of 0.2 M Na_2HPO_4 buffer pH >8, filtered by 0.22 μm CA filter (Corning) and added to the activated nanocages overnight. Nanocages were washed by centrifugation in order to remove the unreacted chemicals and re-suspended in 10 mL of MilliQ water.

Pigment Red 177 (PR177, International Laboratory U.S.A.) was coupled to the 2:NBiAc cages via amidation reaction conducted by phosponium / uranium activation in dimethylformamide (DMF). Briefly, 10 mL of 2:NBiAc cages (acrylic acid moles = 0.0000121) was freeze-dried and resuspended in 10 mL of dimethylformamide (DMF, Sigma-Aldrich). The cage suspension was

purged with nitrogen for 15 min at room temperature under medium stirring. 0.00029 mol of O-benzotriazole-N,N,N',N'-tetramethyl-uronium-hexafluorophosphate (HBTU, Peptides International), 0.00016 mol of N-hydroxybenzotriazole (HOBT, GL Biochem), and 40 μL of N-methylmorpholine (NMM; Fluka Biochemika) were added to the cage suspension and let react at room temperature at medium stirring rate for 5 min under a nitrogen atmosphere. After 5 min, 0.00029 mol of Pigment red 177 (molar ratio of dye/AAc = 10:1) dissolved in DMF was added to the reaction. The reaction was maintained under nitrogen atmosphere for 6 h. Dye coupled cages were washed five times by centrifugation (19,000 rpm, 50 min, 25 °C) with decreasing concentrations of DMF (90%, 75%, 50%, 25%, and 5%) in water in order to eliminate unreacted dye. The cages were re-suspended in 10 mL of water.

Fast Blue B and Safranin O

Fast Blue B (Sigma Aldrich) and Safranin O (Sigma Aldrich) were incorporated into 2:NBiAc nanocages by mixing 10 mL of nanocage suspension with 60 mg/mL dye solution pre-filtered using a nitrocellulose membrane disk filter (0.45 μm pore size). The mixture was allowed to incubate overnight, then washed with a water solution containing 0.1% SDS (five centrifugations, 19,000 rpm, 50 min, 25 °C), and re-suspended in 10 mL of DI water.

Reagent authentication.

The following reagents were obtained through BEI Resources, NIAID, NIH: *Mycobacterium tuberculosis*, Strain H37Rv, Purified Lipoarabinomannan (LAM); NR-14848, Purified lipomannan (LM) nH37Rv, *Mycobacterium tuberculosis*, NR-14850; purified arabinogalactan H37Rv, *Mycobacterium tuberculosis*. CFP-10 Recombinant Protein Reference Standard, NR-49425, ESAT-6, Recombinant Protein Reference Standard, NR-49424, Monoclonal Anti-*Mycobacterium tuberculosis* LAM (produced in vitro), NR-13812, Monoclonal Anti-

Mycobacterium tuberculosis LAM, Clone CS-35 (produced in vitro), NR-13811, Polyclonal Anti-*Mycobacterium tuberculosis* LAM (antiserum, Rabbit), NR-13821, Polyclonal Anti-*Mycobacterium tuberculosis* CFP10 (Gene Rv3874) (antiserum, Rabbit), NR-13801. The following purified pneumococcal polysaccharides were obtained from ATCC: type 23F (US Type 23) (ATCC 25-X), type 9V (US Type 68) (ATCC 253-X), type 14 (US Type 14) (ATCC 23-X), type 6A (US Type 6) (ATCC 14-X™), type 3 (US Type 3) (ATCC 169-X), type 4 (US Type 4) (ATCC 173-X), type 1 (US Type 1) (ATCC 161-X), type 18C (US Type 56) (ATCC 289-X), type 6B (US Type 26) (ATCC 225-X), type 7F (US Type 51) (ATCC 108-X), type 5 (US Type 5) (ATCC 180-X), type 19F (US Type 19) (ATCC 101-X), type 19A (US Type 57) (ATCC 249-X).

Antibody sources and dilutions.

The following antibodies were used for western blot analysis: ESAT6 mouse monoclonal antibody (mAb), Abcam, 1:1000 dilution in blocking buffer (PBS supplemented with 0.2% I-Block and 0.1% Tween 20); CFP10A rabbit polyclonal antibody (pAb), BEI Resources NIAID NIH, 1:50 dilution in blocking buffer; LAM mouse monoclonal antibody (mAb), NR-13811 LAM mAb clone CS-35 BEI Resources NIAID NIH, 1:250 dilution in blocking buffer; INFγ mouse mAb Abcam, 1:1000 dilution in blocking buffer; IL-2 rabbit pAb, Abcam, 1:1000 dilution in blocking buffer; TNFα rabbit pAb, 1:1000 dilution in blocking buffer. Immunoreactivity was revealed by using a specific horseradish peroxidase conjugated anti-IgG secondary antibody (Invitrogen, 1:10,000 dilution in PBS supplemented with 0.2% I-Block and 0.1% Tween 20).

Competition assay to verify specificity of anti LAM mAb clone CS-35.

In order to verify the specificity of band reactivity of the anti-LAM mAb clone CS-35, a competition assay was developed. Prior to staining, the mAb was incubated with a solution

containing excess LAM thus neutralizing and blocking the antibody binding sites on the variable regions. The mAb that was bound to the neutralizing antigen was no longer available to bind to the epitope transferred on the western blot membrane. The blocked mAb and the mAb alone were used to probe duplicate western blots. All other parameters of the western blotting remained the same. The comparison of neutralized mAb to mAb alone showed which staining was specific: the specific staining was absent from the western blot membrane probed with the neutralized mAb. More in detail, 100 µL of anti-LAM antibody (as provided by BEI Resources) was added to 900 µL of 0.2 % I-Block, 0.1 % Tween 20 in PBS and incubated overnight with 250 µL (0.5 mg/mL) of LAM diluted in PBS. In parallel, two aliquots of 150 ng LAM diluted in PBS (L1) and 10 ng LAM diluted in human urine (L2) were separated on a 4-20% Tris Glycine gel (Invitrogen) in the presence of Tris-Glycine SDS running Buffer (Life Technologies) on a Novex X-Cell IITM Mini-Cell (Invitrogen Corporation, USA), at 120 V for 120 minutes. Analytes were transferred onto an Immobilon PVDF membrane (BioRad) for 60 minutes at 50V. The PVDF membrane was separated into two sections containing identical amount of LAM. LAM-saturated and un-modified antibodies were used to probe the PVDF membranes. The membrane was incubated with a peroxidase conjugated goat anti-mouse IgG diluted 1:5,000 in 0.2 % I-Block, 0.1 % Tween 20 in PBS. Three washes of 10 min in 0.2 % I-Block, 0.1 % Tween 20 in PBS were performed. Proteins were detected with an enhanced chemiluminescence system (Supersignal West Dura, Thermo Fischer Scientific) on a Kodak MM4000 Imager.

Urine sample handling prior to analysis

Urinalysis was performed on urine samples using Siemens Multistix 10SG. Urine samples were then centrifuged at 3,700 rcf for 10 minutes at 25 °C to remove cellular debris. Supernatant was

transferred in a new tube. Urine pH was measured and adjusted to 6 with 1M HCl when necessary.

Nanocage incubation with urine samples.

Nanocages (0.1 mL of a 5 mg/mL dry weight water suspension) were mixed with urine samples, let incubate for 15 minutes, separated by centrifugation (16,100 rcf, 25 °C, 10 minutes) and washed 3 times with water. Elution of the nanocages was completed by incubation with 20 μl of 2X sodium dodecyl sulfate (SDS) sample buffer supplemented with 10% 2-mercaptoethanol and heated at 100˚C for five minutes. Cage suspensions were centrifuged (16,100 rcf, 25 °C, 10 minutes); supernatants were saved and subjected to: a) SDS PAGE and silver staining for protein detection, b) SDS PAGE and Pierce™ Glycoprotein Staining Kit (Life Technologies) for LAM detection, c) western blot analysis, or d) immunomacroarray.

Magnetization of Nanocages.

Nanocages (Ø 0.8 μm) were rendered magnetically susceptible by means of an exogenous magnetic label (Fe_3O_4 magnetic nanoparticles (Ø 0.1 μm) coated with oleic acid, Chemicell). Magnetic nanoparticles were incubated with hydrogel nanocages for 15 minutes; the cage suspension was then exposed to neodymium magnets.

Cross link degradation of DHEA containing nanocages.

Cages containing DHEA were subjected to oxidation by $NaIO_4$ solution in order to degrade DHEA cross links. Aliquots of cage suspension were mixed with equivalent volumes of $NaIO_4$ dissolved in 0.05 M citrate buffer pH 5.0 for 10 minutes. DHEA and $NaIO_4$ molar ratio was kept at 1:1.

Cross link degradation of BAC containing nanocages.

1: NBaAl (bis(acryloyl)cystamine, BAC cross-linked) cages were subjected to chemical degradation of cross-linkers in order to increase the effective pore size. 200 µL of 1M dithiothreitol (DTT) was added to 200 µL of 1:NBaAl cage suspension (5 mg/mL) and incubated for 30 minutes. 50 µL of 0.5M iodoacetamide was added to the solution and incubated for 20 minutes in the dark. Cages were centrifuged at 16.1 rcf for 10 minutes. Supernatant was discarded and the pellet was resuspended in 1mL of MilliQ H2O. Washing was repeated for a total of 5 times.

Nanocage incubation with LAM and Anti-LAM antibody

In order to assess the binding availability of dye molecules in the inner volume of the cages, reduced and alkylated cages were incubated with LAM antigen and subsequently with the anti-LAM antibody. LAM (0.16 mg/mL, 10 µL) was spiked in 300 µL of urine. 50 µl of urine with or without LAM was incubated with 50 µL cages before and after degradation. After 30 minutes, cages were separated by centrifugation (16.1 rcf, 10 minutes, 25 °C). Cages were washed with in 1mL of MilliQ H2O, centrifuged at 16.1 rcf for 10 minutes at 25 °C, and re-suspended in a solution of the anti-LAM mAb diluted in PBS (1:20 v/v). After 30 minute incubation at 25 °C, samples were centrifuged at 16,100 rcf for 15 minutes at 25 °C and supernatant was recovered.

Assessing the performance of opened and unopened nanocages through immunomacroarray analysis.

Sample supernatants and anti-LAM mAb were spotted on nitrocellulose membrane and detected with a horse radish peroxidase (HRP) conjugated goat anti-mouse antibody (Invitrogen) and a chemiluminescence detection system (West Dura, Thermo Fischer Scientific). Serial dilutions of samples were performed (1:2-1:4-1:8). The nitrocellulose membrane was allowed to dry and then

blocked with a solution of 0.2% I-Block and 0.1% Tween 20 in PBS for 30 minutes. The membrane was then incubated for 30 minutes with the HRP goat anti-mouse antibody diluted 1:5,000 in 0.2% I-Block, 0.1% Tween 20 in PBS. Three washes of 10 minutes 0.1% Tween 20 in PBS were performed. Proteins were detected with an enhanced chemiluminescence system using a western blot imaging system (Protein Simple).

Single-antibody sandwich immunoassay.

Aliquots of 0.25mL of 3:NBiDAc/TB nanocages (10 mg/mL dry weight water suspension) were incubated with 1 mL of urine containing 1, 0.5, 0.25, 0.125, 0.0625, and 0.03 ng of ESAT-6 for 15 minutes and then centrifuged (16,100 rcf, 25 °C, 5 minutes). The pellet was re-suspended in 0.15 mL MilliQ water and transferred into a 96-well microtiter plate. Cages were washed with PBS (centrifugation parameters: 3,800 rcf, 25 °C, 5 minutes), incubated with 0.2 mL of 0.1M $NaIO_4$ (Sigma) dissolved in 0.05 M citrate buffer pH 5.0 for 10 minutes, washed with PBS (centrifugation parameters: 3,800 rcf, 25 °C, 5 minutes), and re-suspended in 0.15 mL of PBS. Cages and wells were blocked with 0.2 mL PBS supplemented with 0.4% I-Block, 5% PEG 8000, and 0.1% Tween 20 for 1 hour at room temperature on a rocker. Anti ESAT-6 mouse mAb, Abcam, was HRP labeled using the Lightning-Link HRP conjugation Kit (Innova Biosciences) according to vendor instructions, added to the wells at a 1:2000 dilution in PBS supplemented with 0.4% I-Block and 0.1% Tween 20, and let incubate overnight at 4 °C on a rocker. The plate was washed 3 times with 0.2 mL of wash buffer (PBS supplemented with 0.05% v/v Tween 20). Colorimetric signal was generated with 0.1 mL of 3,3’,5,5’-tetramethylbenzidine (TMB Substrate, Thermo, 15 minutes in the dark) plus 0.05 mL of 0.25N HCl and read at 450 nm using Multiskan plus (Fisher Scientific) plate reader.

Nanocage-integrated lateral flow immunoassay.

Anti ESAT6 mouse mAb (Abcam) was diluted 1:10 in PBS for a final concentration of 0.1 mg/mL. 0.01 mL of the diluted mAb solution was deposited on a glass fiber membrane filter (1 × 4 cm, Millipore) and allowed to dry at 37°C in a forced air oven (Fisher scientific Isotemp). Glass fiber membrane was incubated with 10 mL of blocking solution (50 mg/mL PEG 8000 in PBS) for 30 minutes at room temperature on a rocker. The membrane was rinsed in washing buffer (PBS supplemented with 0.05% v/v Tween 20) and let dry at 37°C in a forced air oven. Nanocages (3:NBiDAc and TB, **Table 7**, 0.5 mL of 10 mg/mL dry weight water suspension) were mixed with 10 mL of human urine containing 1-0.03 ng of ESAT6 and incubated for 15 minutes. Cages were washed twice with PBS and incubated with 0.02 mL of 0.1M $NaIO_4$ dissolved in 0.05 M citrate buffer pH 5.0 for 5 minutes. This step caused the cages to shift shape and display the captured antigen. Cages were then centrifuged (16,100 rcf, 25 °C, 5 minutes) and re-suspended in 1 mL of PBS. Two wicks (1.5 × 2.5 cm, extra thick filter paper, Bio-Rad) were placed on the extremities of the glass fiber membrane filter obtained as previously described. The cage suspension was deposited on one of the wicks and let flow for 5 minutes. Cages containing the ESAT6 antigen arrested on the line. Cages were imaged with a scanner. HRP labelled anti ESAT6 mAb diluted 1:100 in PBS was allowed to flow through the membrane. The membrane was then incubated with SuperSignal Chemiluminescence substrate and the signal was detected with a Kodak imager.

Saccharide quantification.

Total saccharide concentration of the LAM reference standard (BEI Resources) was quantified by the Anthrone method. D-(+)-Glucose (Sigma-Aldrich) was used as calibrator (0.1 mg/mL, 0.08 mg/mL, 0.06 mg/mL, 0.04 mg/mL, 0.02 mg/mL, 0 mg/mL). 50 μL of calibrators and

samples were added to 100 μL of chilled 75% H_2SO_4 solutions and to 200 μL of Anthrone solution (100 mg/mL in ethanol). Samples were placed at 100 °C for 15 minutes and then in ice. Absorbance of samples was read at λ=630 nm with a UV-2501 PC spectrophotometer (Shimadzu).

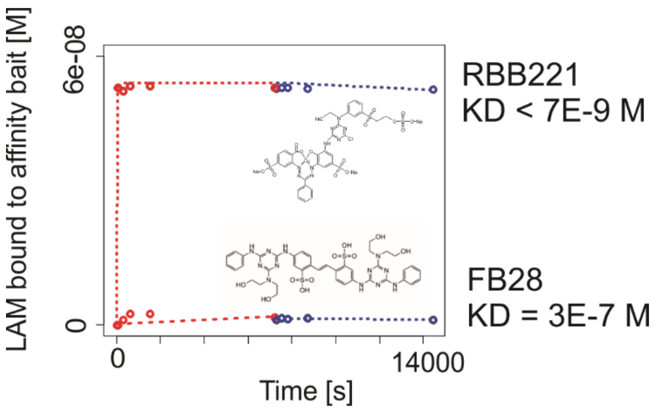

Fig. S1. The K_d affinity between RB221 and LAM exceeds that of FB28. The equilibrium dissociation constant K_d was obtained via binding kinetic assays. Association (red) and dissociation (blue) curves were experimentally determined by incubating RB221 and FB28 functionalized nanocages with LAM diluted in PBS. Cages were precipitated by centrifugation and supernatants were quantified via immunomacroarray analysis. Bound LAM [LB] was measured at 0, 60, 300, 600, 1500, 7200 seconds for the association kinetics and at 0, 60, 300, 600, 1500, and 7200 seconds for the dissociation kinetics.

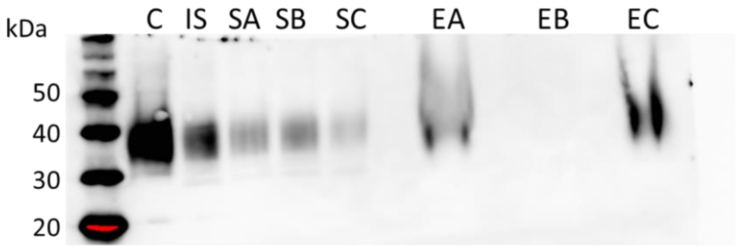

Fig. S2. Copper dyes outperform copper free dyes such as fast blue B and safranin O. Nanocages functionalized with Fast Blue B salt and Safranin O did not capture all solution phase LAM spiked in human urine (SA, SB, and SC). The affinity of the copper free dyes towards LAM is therefore inferior compared to RB221, which captured all LAM in solution as evidenced by the absence of detectable signal in the supernatant (Fig. 1, main text). 100 ng LAM were spiked in 200 μL urine and incubated with 100 μL nanocages (A=Fast Blue B, B = Safranin O, and C = Cibacron Brilliant Blue F3GA). Captured LAM was eluted with 5% sodium deoxycholate in water (EA, EB, and EC).

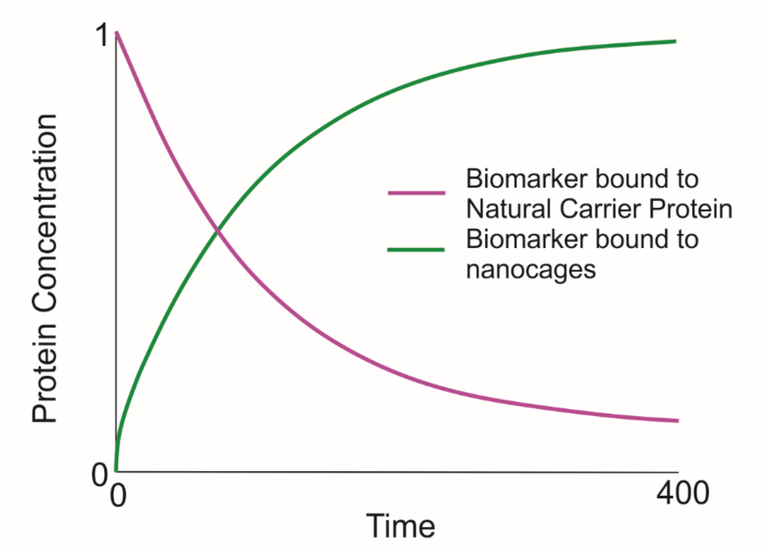

Fig. S3. Nanocages dissociate biomarker from interfering substances, in silico mathematical modeling. The modeling in section “Theoretical justification for affinity capture and sensitivity” shows that the greater the affinity of the TB antigens for the chemical bait incorporated in the nanocages, the higher the proportion of TB antigen dissociated from urinary carrier proteins and captured in the nanocages. With adequate high affinity binding, such as the one demonstrated by the RB221 dye, effectively all LAM analyte is sequestered into the nanocage (including any LAM bound to carrier proteins).

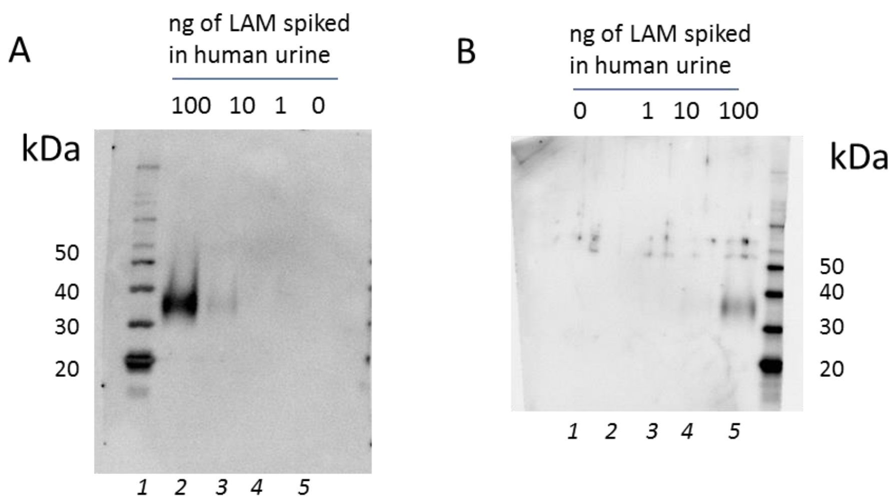

Fig. S4. CS-35 mAb is specific for LAM diluted in human urine, batch verification. A. Absence of background bands in urine pre-treated with the nanocages is noted using western blot analysis and CS-35 mAb. Lane 1: molecular weight marker; lanes 2-4: 100, 20, and 1 ng LAM in urine, respectively; lane 5 urine in absence of LAM. **B.** Urine pre-treated with the nanocages was analyzed by means of western blot and CS-40 polyclonal anti LAM antibody. Lane 1: urine in absence of LAM; lanes 3-5: 1, 10, and 100 ng LAM in urine, respectively.

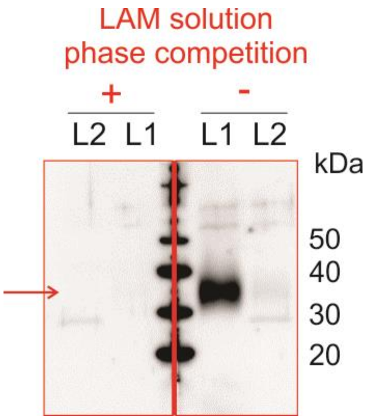

Fig. S5. Competition assay confirmed the specificity of CS-35 mAb. L1 = 150 ng LAM in PBS, L2 = 10 ng LAM in human urine.

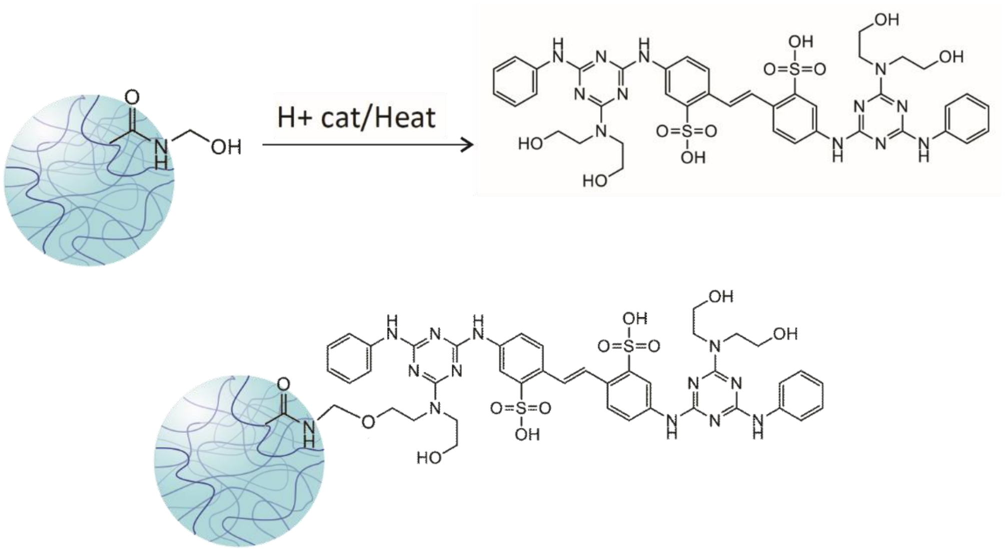

Fig. S6. Coupling chemistry to covalently incorporate the FB28 dye in the inner volume of the nanocages.

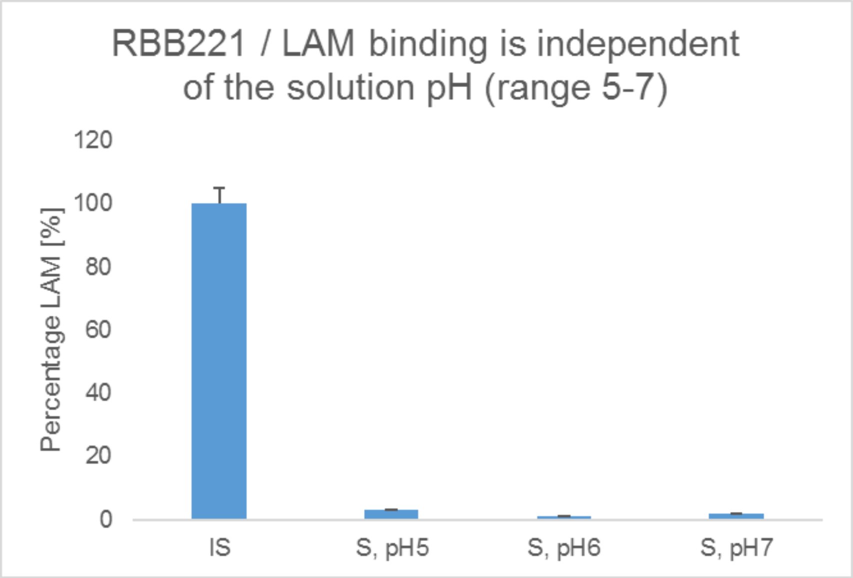

Fig. S7. LAM binding to RB221 and depletion from supernatant are independent of pH in a 5 to 7 range. At all relevant pH tested, LAM was depleted from the supernatant. Nanocages sequestration of LAM was characterized at three different pH values: 5, 6, and 7. Cages were incubated with a 1.5 ng/μL solution of LAM diluted in 50 mM citrate buffer pH 5, 50 mM citrate buffer pH 6, and phosphate buffer saline pH 7. Initial solution and supernatants were analyzed with immune macroarray assay; the deriving signal was quantified with ImageJ software. The barplot reports % of LAM present in the initial solution (IS, 100%) and % of LAM detected in the supernatant after cage sequestration at pH5 (S, pH5), pH6 (S, pH6), and pH7 (S, pH7).

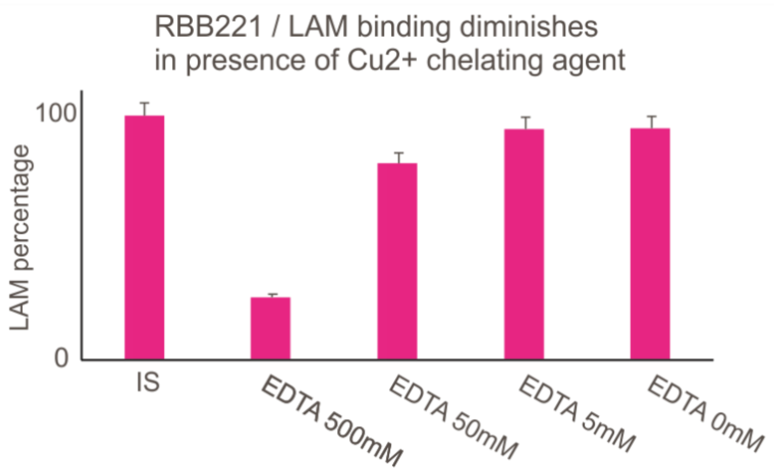

Fig. S8. RB221 binding to LAM is hindered by the presence of a copper-chelating agent (EDTA). Cages were treated with EDTA at the following concentrations: 500 mM, 50 mM, 5 mM, and 0mM. Cages were incubated with a 1.5 ng/µL solution of LAM diluted in phosphate buffer saline pH 7. Initial solutions and supernatants were analyzed with immune macroarray assay; the deriving signal was quantified with ImageJ software. The barplot reports % of LAM present in the initial solution (IS, 100%) and % of LAM contained in the cages treated with 500 mM EDTA, 50 mM EDTA, 5 mM EDTA, and 0 mM EDTA.

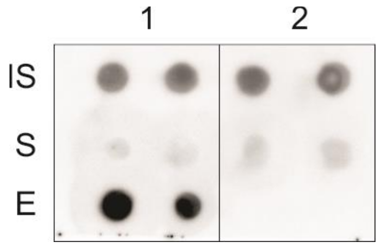

Fig. S9. RB221-LAM interaction requires intact diol moieties of LAM as proven by $NaIO_4$ oxidation. Nanocages were incubated with 200 μl of urine containing 10 ng LAM (IS = initial solution, S = supernatant). Elution via chemical degradation of LAM was achieved by adding $NaIO_4$ to the nanocages to a final concentration of 50 mM pH 5. After incubation for 15 minutes in ice, the cages were spinned down and the supernatant was spotted on a PVDF membrane (E1). LAM was successfully eluted from the cages and its antibody binding site was intact. Stronger oxidation conditions (100 mM $NaIO_4$ pH 9 1 hour at room temperature) caused a more extensive diol bond oxidation and loss of antibody binding site (E2).

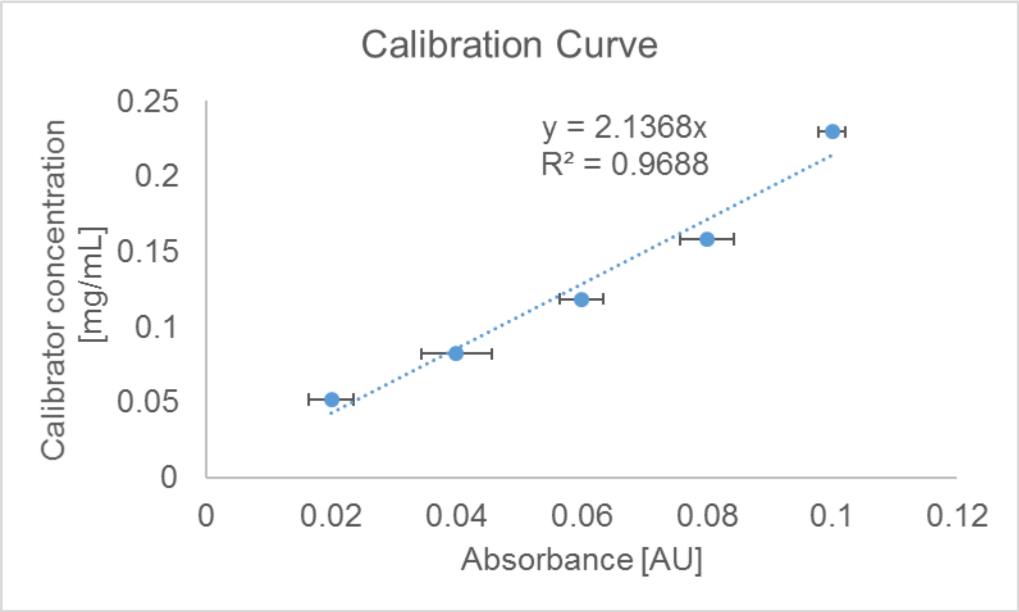

Fig. S10. Carbohydrate concentration in the LAM reference standard (0.160 mg/ml) was quantified by a linear colorimetric assay. The Anthrone method (supplementary materials and methods) was applied and glucose was used as calibrator. The calibration curve demonstrates the assay is linear in the tested range.

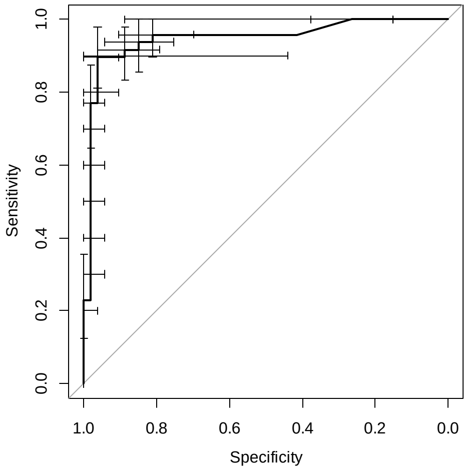

Fig. S11. Plot of the 95% CI of the sensitivity and specificity of the ROC analysis reported in Fig. 3C. The CI were computed with 2000 stratified bootstrap replicates.

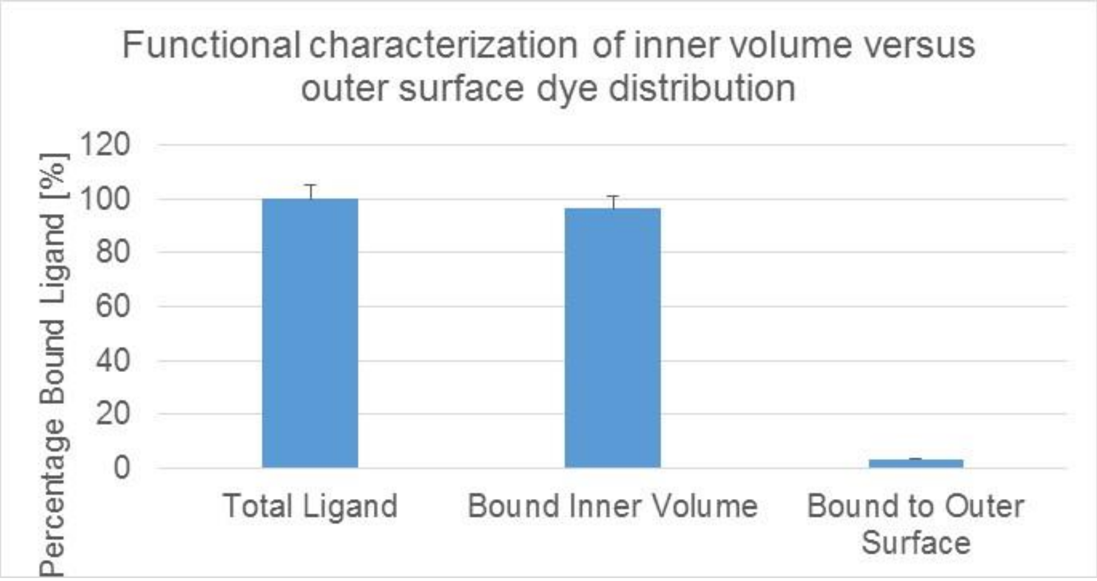

Fig. S12. The RB221 dye is immobilized in the inner volume of the cages and is available for high–molecular weight ligand binding after cross-link degradation and consequent increase of the effective pore size. RB221 functionalized cages were incubated with the LAM antigen and subsequently with the CS-35 anti-LAM monoclonal antibody (MW~150,000). Nanocages that were subjected to cross link degradation exhibited an increased amount of antibody bound to the inner volume of the cage, more than 40-fold with respect to non-degraded cages.

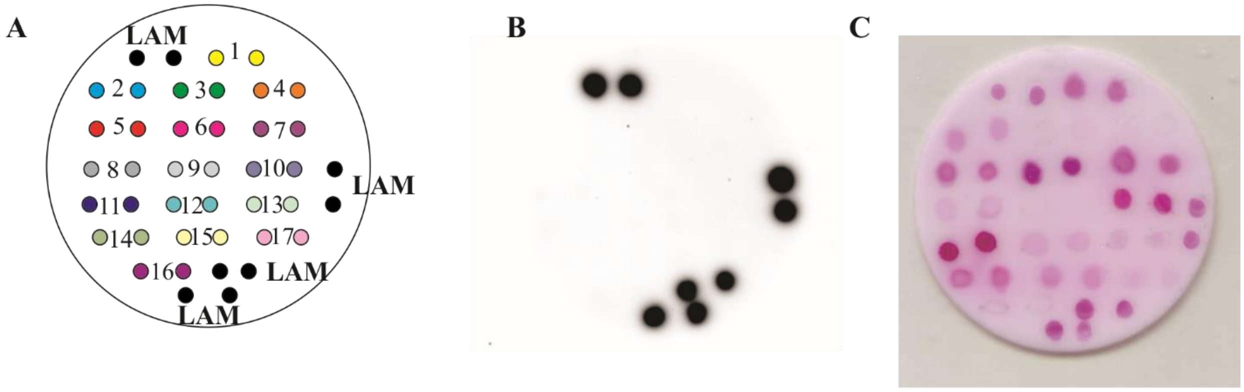

Fig. S13. CS-35 anti-LAM mAb does not cross-react with purified polysaccharides from *N. meningitidis* and *S. pneumoniae*. A. Layout of the immunomacroarray. Purified polysaccharides (1 ng) from the following pathogens were spotted on a PVDF membrane: 1. *Neisseria meningitidis* serogroup W-135; 2. *Neisseria meningitidis* serogroup Y; 3. *Neisseria meningitidis* serogroup A; 4. *Neisseria meningitidis* serogroup C; 5. *Streptococcus pneumoniae* serotype 18C; 6. *Streptococcus pneumoniae* serotype 23F; 7. *Streptococcus pneumoniae* serotype 6B; 8. *Streptococcus pneumoniae* serotype 9V; 9. *Streptococcus pneumoniae* serotype 4; 10. *Streptococcus pneumoniae* serotype 14; 11. *Streptococcus pneumoniae* serotype 7F; 12. *Streptococcus pneumoniae* serotype 5; 13. *Streptococcus pneumoniae* serotype 3; 14. *Streptococcus pneumoniae* serotype 1; 15. *Streptococcus pneumoniae* serotype 6A ;16. *Streptococcus pneumoniae* serotype 19F; 17. *Streptococcus pneumoniae* serotype 19A; LAM from *Mycobacterium tuberculosis*. The membrane was stained using CS-35 mAb as described in the Methods section. **B.** The CS-35 anti-LAM mAb does not interact with polysaccharides from non-*Mycobacterium tuberculosis* antigens as demonstrated by immunomacroarray analysis. **C.** Periodic acid–Schiff stain of the polysaccharides spotted on a PVDF membrane following the layout in panel A.

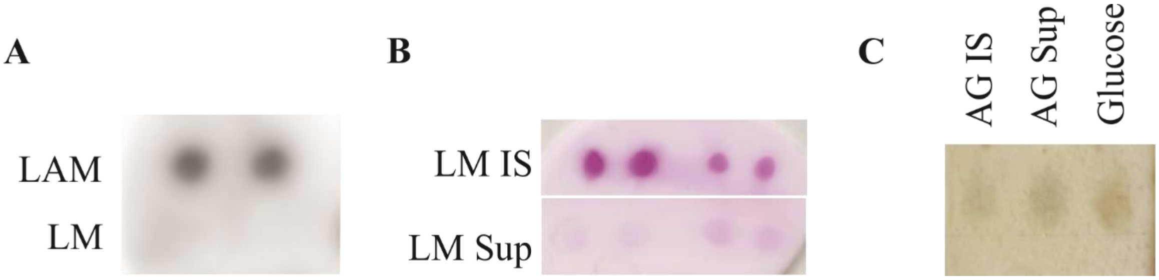

Fig. S14. Nanocage capturing followed by CS-35 antibody detection is specific for LAM and does not cross-react with *M. tuberculosis* lipomannan and arabinogalactan. A. The CS-35 anti-LAM monoclonal antibody does not cross-react with *Mycobacterium tuberculosis* lipomannan, as demonstrated by immunomacroarray analysis. One nanogram of purified LAM and lipomannan (BEI Resources) were deposited on a PVDF membrane and detected as described in the Methods section. **B.** RB221 nanocages capture *Mycobacterium tuberculosis* lipomannan from solution. Solutions of lipomannan in PBS was prepared at concentration of 1 and 0.5 mg/mL. 50 μL of RB221 nanocage suspension (5 mg/mL) were mixed with 50 μL of lipomannan solution and allowed to incubate for 30 minutes at room temperature. The supernatant was separated by centrifugation (10 minutes, 25C, 16.1 rcf). The initial solutions (IS) and the supernatants (Sup) were deposited on a PVDF membrane and stained using the periodic acid–Schiff method. **C.** RB221 nanocages do not capture *Mycobacterium tuberculosis* arabinogalactan from solution. Purified arabinogalactan (BEI Resources) was dissolved in DMSO and diluted with DI water to a concentration of 1 mg/mL. 50 μL of RB221 nanocage suspension (5 mg/mL) were mixed with 50 μL of arabinogalactan solution and allowed to incubate for 30 minutes at room temperature. The supernatant was separated by centrifugation (10 minutes, 25C, 16.1 rcf). The initial solution (IS, 2 μL), the supernatant (Sup, 2 μL), and a positive control (glucose, 2 μg) were deposited on a silica gel thin layer chromatography sheet (J.T. Baker) and dried in a convection oven (110°C, 45 minutes). The sheet was submerged in

anthron solution (0.05 g anthron, 0.5 mL ethanol, 25 mL of sulfuric acid) and placed in the oven (100°C, 30 minutes). The initial solution and the supernatant after nanocages incubation have similar signal demonstrating that the arabinogalactan is not captured by the nanocages.

Table S1. Nanocage bait chemistries screened to capture and enrich LAM from human urine.

Number	Dye name
1	Trypan Blue
2	Cibacron Blue
3	Bismark Brown
4	Acid blue 3
5	Acid Blue 9
6	Acid red 92
7	Pyranine
8	Fast green FCF
9	Erythrosin b
10	Evans Blue
11	Evans Black
12	Diamine Green
13	Acid Black 48
14	Toluidine Blue O
15	Cibacron Blue + Vinyl Sulfonic acid
16	Allylamine
17	Remazol Brillian Blue R
18	Red 9
19	Disperse Yellow 3
20	Congo Red
21	Safranin O
22	Vinyl Phenyl Boronic Acid
23	Alcian Blue
24	Carmine
25	Alizarin Red 5
26	Alizarin Cyanin
27	Gallein
28	Haematoxylin
29	Alizarin Black B
30	Solvent Blue 67
31	Fast Blue Salt B
32	Benzylviologel dichloride
33	Solvent Blue 70
34	Rutin Hydrate
35	Nitrotetrazolium blue chloride
36	Reactive blue 221
37	Fluorescent brightener 28

Table S2. Medical characteristics of diseased TB-negative controls.

Patient ID	Medical condition	CD4+ count	viral load	HIV
CBBA 49	Genitourinary infection, chronic gastroenteritis, giardiasis, hypokalemia	4	8646	+
CBBA 56	Pneumonia, cardiomyopathy, type 2 diabetes mellitus, cryptosporidiosis			+
CBBA 70	Lung cancer (not subtyped)	64	1696568	+
CBBA 99	"Community acquired pneumonia", anemia, hyperbilirubinemia, AIDS	108	193283	+
CBBA 182	Genitourinary infection	254	1310	+
CBBA 192	Sepsis, urinary infection, gastrointestinal cancer, intestinal obstruction, herpes zoster infection, hemiparesis	8	4698	+
CBBA 209	Moderate cachexia, gastroenteritis, HIV respiratory syndrome	130	34000	+
SDJ 63	Pneumonia (not subtyped)	281	40	+
SDJ 114	HIV respiratory syndrome	196		+
SDJ 132	Pneumonia, left pleural effusion	359		+
SDJ 134	Gastroenteritis, acute pyelonephritis, moderate anemia	265		+
SDJ 150				+
234	Cough, fever	258	62245	+
1040	Hemoptysis			-

Table S3. Urinalysis results for all study participants.

	Leukocyte esterase	Nitrites	Urobilin ogen	Protein	pH	Blood	Specific gravity	Ketone	Bilirubin	Glucose
216	N	N	0.2	N	6	N	1.02	N	N	N
217	N	N	0.2	N	5	N	1.015	N	N	N
226	N	N	0.2	N	6.5	N	1.005	N	N	N
227	N	N	0.2	N	5	N	1.025	N	N	N
234	+++	N	0.2	+++	7	+++	1.01	N	+	N
237	N	N	0.2	N	7	N	1.01	N	N	N
1001	N	N	0.2	N	6	N	1.005	N	N	N
1003	N	N	0.2	N	5	N	1.025	N	N	N
1009	N	N	0.2	N	8.5	N	1	N	N	N
1014	N	N	0.2	N	5	N	1.015	N	++	N
1017	N	N	0.2	N	6.5	N	1.015	N	N	N
1022	N	N	0.2	N	5	N	1.02	N	++	N
1025	N	N	0.2	N	7	N	1.01	N	N	N
1029	N	N	0.2	N	6.5	N	1.005	N	N	N
1035	N	N	0.2	N	6.5	N	1.02	N	N	N
1041	N	N	0.2	N	7	N	1.01	N	N	N
1043	N	N	0.2	N	6	N	1.025	N	N	N
1045	N	N	0.2	N	6.5	+++	1.015	N	N	N
1048	N	N	0.2	N	6	N	1.01	N	N	N
1051	N	N	0.2	N	5	N	1.02	N	N	N
1055	N	N	0.2	N	5	+++	1.015	N	N	N
1058	N	N	0.2	N	6	N	1.005	N	N	N
1060	N	N	0.2	N	6.5	+++	1.01	N	N	N
1062	N	N	0.2	N	7.5	N	1.005	N	N	N
1064	N	N	0.2	N	6	N	1.015	N	N	N
1065	N	N	0.2	N	6.5	N	1.02	N	+	N
1066	N	N	0.2	N	6.5	N	1.01	15	++	N
1067	N	N	0.2	N	7.5	N	1	N	N	N
1072	N	N	0.2	N	6.5	N	1.005	N	N	N
1073	+	N	0.2	N	7	N	1.01	N	+	N
1074	+	N	0.2	N	7	HEMOLIZE D TRACE	1.01	N	N	N
1075	N	N	0.2	N	7	N	1.005		+	N
1079	N	N	0.2	N	5	N	1.03	N	+	N
1080	N	N	0.2	N	6.5	N	1.01	N	N	N
1082	+	N	0.2	N	7	N	1.015	N	N	N
1083	N	N	0.2	N	5	N	1.015	N	N	N
1088	N	N	0.2	N	6.5	HEMOLIZE D TRACE	1.015	N	+++	N
1090	N	N	0.2	N	7	N	1.005	N	N	N
1093	N	N	0.2	N	6.5	N	1.005	N	++	N
1096	N	N	0.2	N	7.5	N	1.005	N	N	N
1097	N	N	0.2	N	6.5	HEMOLIZE D TRACE	1.015	N	N	N
1100	N	N	0.2	N	7	N	1.005	N	N	N
1101	N	N	0.2	N	7	N	1.025	5	N	N
1102	N	N	0.2	N	5	N	1.025	N	N	N
1104	N	N	0.2	N	6.5	N	1.025	N	N	N
1106	N	N	0.2	N	6.5	hemolized trace	1.02	N	+++	N
3000	N	N	0.2	N	6.5	N	1.01	N	N	N
3006	N	N	0.2	N	7	N	1.01	N	N	N
4002	N	N	0.2	N	7	N	1.005	N	N	N
49	+	N	0.2	N	6.5	N	1.015	N	N	N
56	N	N	0.2	N	6	N	1.0100	N	N	N

70	N	N	0.2	N	6	N	1.02	5	+	N
99	N	N	0.2	N	6.5	+++	1.0150	N	N	N
182	+	N	0.2	N	8	N	1.0050	N	N	N
192	N	N	0.2	N	8.5	N	1.01	40	N	N
209	N	N	0.2	N	6	N	1.0150	N	N	N
63	N	N	0.2	N	6.5	N	1.0050	N	+	N
114	N	N	0.2	N	5	N	1.03	N	N	N
132	N	N	0.2	N	7	N	1.0100	trace	N	N
134	N	N	0.2	N	6	N	1	5	N	N
150	N	N	0.2	N	6	Trace	1.0100	N	N	N
1040	N	N	0.2	N	5	N	1.02	5	N	N
5640	N	N	0.2	N	5	N	1.0150	N	N	N
2002	N	N	0.2	N	6	N	1.0100	N	N	N
2008	N	N	0.2	N	6.5	N	1.0150	N	N	N
2615	N	N	0.2	N	5	N	1.0050	N	N	N
2639	N	N	0.2	N	6	N	1.0050	N	N	N
2774	N	N	0.2	N	6	N	1.0100	N	N	N
5626	N	N	0.2	N	6.5	N	1.0100	N	N	N
2808	N	N	0.2	N	5	N	1.0050	N	N	N
2835	N	N	0.2	N	5	N	1.0150	N	N	N
2855	N	N	0.2	N	5	N	1.0200	N	N	N
2860	N	N	0.2	N	6	N	1.0100	N	N	N
2861	N	N	0.2	N	6.5	N	1.0100	N	N	N
2873	N	N	0.2	N	6	N	1.0100	N	N	N
2877	N	N	0.2	N	6	N	1.0150	N	N	N
2878	N	N	0.2	N	6	N	1.0150	N	N	N
2900	N	N	0.2	N	5	N	1.0100	N	N	N
5002	N	N	0.2	N	5	N	1.0100	N	N	N
5013	N	N	0.2	N	5	N	1.0100	N	N	N
5014	N	N	0.2	N	6.5	N	1.0150	N	N	N
5332	N	N	0.2	N	5	N	1.0150	N	N	N
5871	N	N	0.2	N	6	N	1.0050	N	N	N
5872	N	N	0.2	N	6	N	1.0150	N	N	N
6047	N	N	0.2	N	6	N	1.0150	N	N	N
5890	N	N	0.2	N	6	N	1.0150	N	N	N
5891	N	N	0.2	N	6	N	1.0200	N	N	N
6034	N	N	0.2	N	6	N	1.0150	N	N	N
5868	N	N	0.2	N	6.5	N	1.0150	N	N	N
6035	N	N	0.2	N	6.5	N	1.0150	N	N	N
5667	N	N	0.2	N	6	N	1.0100	N	N	N
6036	N	N	0.2	N	6	N	1.0100	N	N	N
6037	N	N	0.2	N	5	N	1.0050	N	N	N
6038	N	N	0.2	N	5	N	1.0100	N	N	N
6042	N	N	0.2	N	5	N	1.0100	N	N	N
6039	N	N	0.2	N	5	N	1.0150	N	N	N
6040	N	N	0.2	N	6	N	1.0050	N	N	N
5651	N	N	0.2	N	6	N	1.0150	N	N	N
6041	N	N	0.2	N	6	N	1.0050	N	N	N
6043	N	N	0.2	N	5	N	1.0100	N	N	N
6044	N	N	0.2	N	5	N	1.0050	N	N	N
